# Can Bengal Tiger (*Panthera tigris tigris*) endure the future climate and land use change scenario in the East Himalayan Region? Perspective from a multiple model framework

**DOI:** 10.1002/ece3.10340

**Published:** 2023-08-06

**Authors:** Jyotish Ranjan Deka, Sk. Zeeshan Ali, Mujahid Ahamad, Priyanka Borah, Govindan Veeraswami Gopi, Ruchi Badola, Rabindra Sharma, Syed Ainul Hussain

**Affiliations:** ^1^ Wildlife Institute of India Dehradun Uttarakhand India; ^2^ Kaziranga National Park and Tiger Reserve Assam India

**Keywords:** climate change, conservation planning, indo‐Burma biodiversity hotspots, large mammal, protected areas threat, Tiger habitat

## Abstract

Large mammals are susceptible to land use and climate change, unless they are safeguarded within large, protected areas. It is crucial to comprehend the effects of these changes on mammals to develop a conservation plan. We identified ecological hotspots that can sustain an ecosystem for the endangered Bengal tiger (*Panthera tigris tigris*), an umbrella species. We developed three distinct ensemble species distribution models (SDMs) for the Bengal tiger in the Indian East Himalayan Region (IEHR). The first model served as the baseline and considered habitat type, climate, land cover, and anthropogenic threats. The second model focused on climate, land use, and anthropogenic threats, the third model focused on climate variables. We projected the second and third models onto two future climate scenarios: RCP 4.5 and RCP 8.5. We evaluated the threats possess to protected areas within eco‐sensitive zone based on the potential tiger habitat. Finally, we compared the potential habitat with the IUCN tiger range. Our study revealed that the Brahmaputra valley will serve as the primary habitat for tigers in the future. However, considering the projected severe climate scenarios, it is anticipated that tigers will undergo a range shift towards the north and east, especially in high‐altitude regions. Very high conservation priority areas, which make up 3.4% of the total area, are predominantly located in the riverine corridor of Assam. High conservation priority areas, which make up 5.5% of total area are located in Assam and Arunachal Pradesh. It is important to note that conservation priority areas outside of protected areas pose a greater threat to tigers. We recommend reassessing the IUCN Red List's assigned range map for tigers in the IEHR, as it is over‐predicted. Our study has led us to conclude both land use and climate change possess threats to the future habitat of tigers. The outcomes of our study will provide crucial information on identifying habitat hotspots and facilitate appropriate conservation planning efforts.

## Graphical abstract

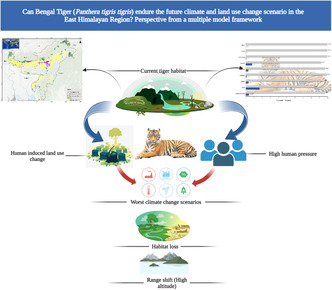

## 1 INTRODUCTION

Climate change significantly affects the distribution of wildlife, leading to changes in species' niche ecosystems, ranges, and biodiversity (Bellard et al., 2012). In the coming decades, it will lead to the extinction of species, habitat degradation, and have a severe impact on tangible and intangible ecosystem services and livelihood opportunities (Newbold, 2018). Furthermore, human‐induced land use activities, particularly around protected areas, have also resulted in a biodiversity crisis (Pekin & Pijanowski, 2012; Sieber et al., 2015). The combined impact of climate and land use changes can have a strong effect on wildlife populations, typically through habitat loss and degradation, which are emerging as a serious threat to wildlife (Newbold, 2018; Sieber et al., 2015).

Recent observations indicate that anthropogenic‐induced climate change and land transformation are posing a global threat to wildlife (Thuiller et al., 2006). In Southeast Asia, the combined impact of climate and land use change is having a negative effect on mammalian habitats, highlighting the urgent need for a worldwide shift towards sustainability (Baisero et al., 2020; Brodie, 2016). Hunting, and land use change are responsible for an average of 41% reduction in the distribution of tropical mammalian species (Gallego‐Zamorano et al., 2020). The tiger, an endangered species on the International Union for Conservation of Nature's (IUCN) red list, is among the mammalian fauna threatened by land use change, climate change and hunting in the tropical region (Deb et al., 2019; Mukul et al., 2019; Penjor, Kaszta, et al., 2021; Penjor, Wangdi, et al., 2021; Thapa et al., 2022; Trisurat et al., 2015; Velho et al., 2012).

The Bengal tiger (*Panthera tigris tigris*) is a sub‐species of *Panthera tigris* found in Bangladesh, Bhutan, India, Nepal and Myanmar (Goodrich et al., 2022). Their habitats vary from dry to wet deciduous forests, evergreen to mangrove forests and terai grasslands to mixed conifer‐broadleaf forests (Jhala et al., 2011). In India, their population is predominantly found in protected areas (PAs), and disturbances or lack of connectivity in the corridors isolate them from one PA to another. Despite threats from human‐altered landscapes, conservation policies, wildlife trafficking control and strengthened conservation efforts by the Indian government have resulted in an increasing tiger population (Jhala et al., 2020). However, due to their limited habitat and direct or indirect human intervention with increasing tiger populations, there is a potential for increase of human–tiger conflicts in the near future. Furthermore, the destruction of habitats and the loss of connectivity among tiger populations have led to inbreeding, which has caused colour aberrations in some tigers, resulting in the appearance of golden tigers in Kaziranga, as reported by Sharma (2014). Thus, it is necessary to assess the species' sensitivity on a large spatial scale and evaluate its management (Thuiller et al., 2006). Developing adaptive management strategies supported by a suitability model that considers the effects of climate and land use could help to conserve or minimize the loss of tiger habitat.

Predictive modelling and mapping of species distribution are commonly used to understand their topographical distribution. Recent advances in species distribution models (SDMs) enable us to forecast the effects of human activity on biodiversity patterns at various spatial scales (Guisan & Thuiller, 2005). Accurate SDMs are required to make informed nature policy decisions regarding species conservation (Thomaes et al., 2008), including identifying the necessary environmental conditions for a species (Peterson et al., 2011) and predicting their geographic range to understand their ecological requirements (Duan et al., 2014). However, SDMs often have limited understanding of the combined effects of land use, climate and anthropogenic disturbances. To address this, a multiple model framework was developed to determine the potential habitat of tigers. The framework incorporates three independent models to improve understanding of uncertainties in the modelling process.

The Indian East Himalayan Region (IEHR), being geographically isolated from the rest of India, harbours the most distinct tiger population (Armstrong et al., 2021). In India, the tigers across their entire ranges, including the IEHR, face threats from both anthropogenic activity and climate change (Ripple et al., 2014). Recent studies have shown that mammal assemblages in the Himalayan landscape of Bhutan and the Indian Himalayan region are negatively impacted by climate and land use change (Tiwari, 2000; Penjor, Kaszta, et al., 2021; Penjor, Wangdi, et al., 2021). In the context of India, numerous studies have specifically examined the potential habitat of tigers and the impact of climate change (Bajaj & Amali, 2019; Karwariya et al., 2017; Prajapati et al., 2014; Rather et al., 2020; Sarkar et al., 2017; Singh et al., 2009; Sinha et al., 2012). However, no observations have specifically assessed the impact of these changes on tiger populations in the IEHR.

To address this research gap, this study aims to determine the potential habitat of tigers in the IEHR and evaluate changes in their future distribution caused by climate change and land use. Additionally, the study evaluates the threats faced by PAs with the objective of informing efforts to prioritize conservation. The study employs SDM with a multiple model framework to predict the potential tiger habitat in the IEHR. The SDM is validated using the IUCN tiger range in the region. The study's findings could provide critical information for conservation efforts aimed at protecting the tiger population and establishing human–carnivore coexistence.

## 2 METHODS

### 2.1 Study area

**FIGURE 1:**
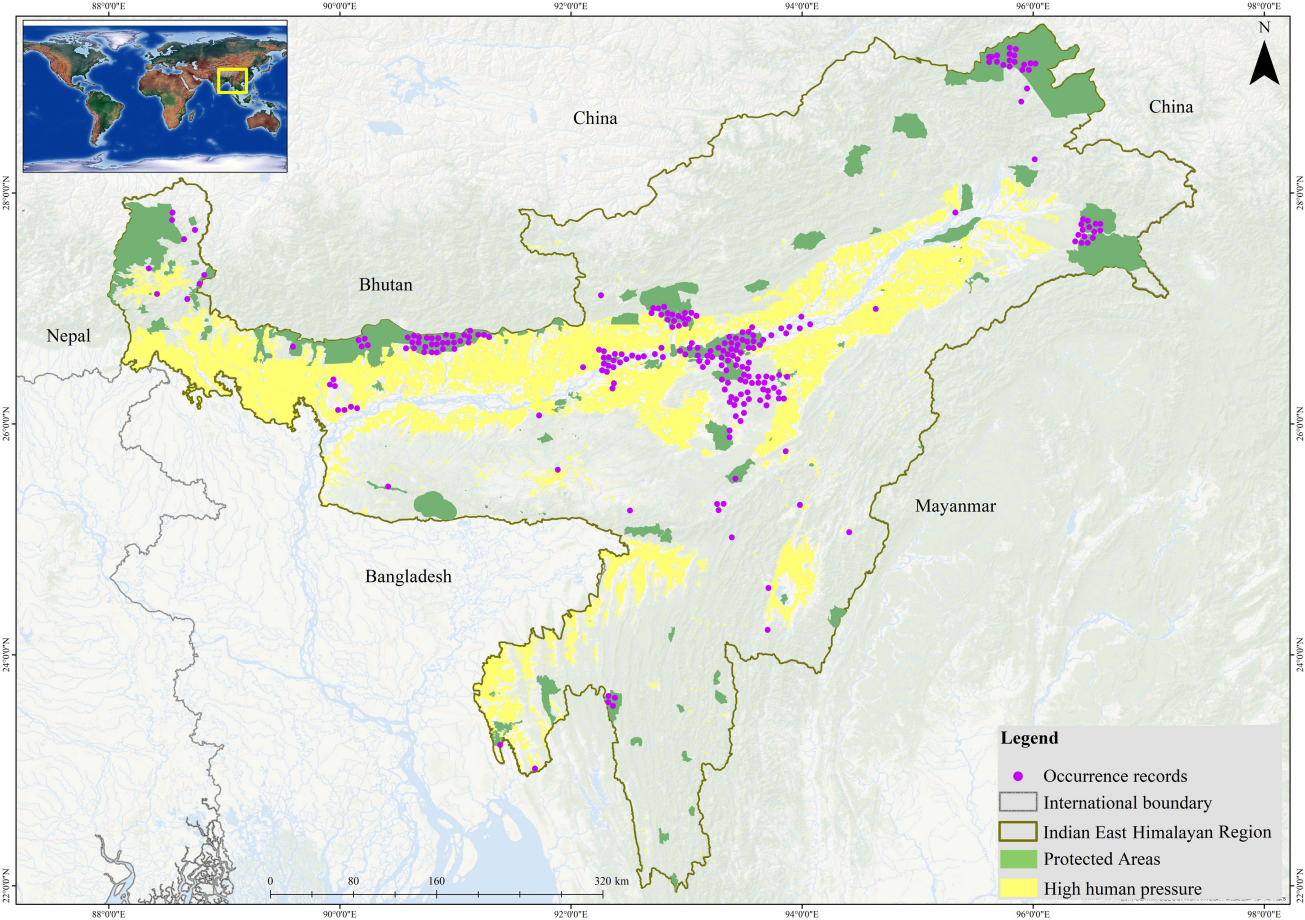
Indian East Himalayan Region with the tiger occurrences and the human pressure experienced by this region. High human pressure areas were shown in the light‐yellow colour along with the protected areas in green colour.

The IEHR comprises Arunachal Pradesh, Assam, Manipur, Meghalaya, Mizoram, Nagaland, Sikkim, Tripura and North Bengal with a total geographical area of 273,490.36 km^2^ (Figure 1), which is almost 8% of the country's total geographical area. The region extends from 21°56′26.296″ East to 29°27′41.59″ East latitude and 87°59′14.024″ North to 97°24′43.345″ North longitude. The region is the confluence of two biodiversity hotspots, viz. Indo‐Myanmar and the Himalayas (Myers et al., 2000). It is considered the junction of two bio‐geographic realms, the Indo‐Malayan and the Palaearctic, which support the most endangered and endemic species in the landscape. The landscape is composite of hills, mountains, river, alpine forest, evergreen forest and islands which, lies in the greater Brahmaputra, Dibang, Tista and Barak valleys. The area primarily comprises tropical forest (less than 1000 m), subtropical forest (1000–2000 m), temperate forest (2000–3500 m), subalpine forest (3500–4500 m) and alpine forest (4500–5000 m) (Champion & Seth, 1968).

The IEHR sustains diverse communities and livelihoods through agriculture, tourism and natural resource extraction (Bawa et al., 2007). Subsistence farming and cash crops are pivotal, while captivating landscapes and cultural heritage attract tourists. However, it is essential to strike a balance between development and biodiversity conservation, considering the rapid extraction of natural resources, for the long‐term socio‐economic well‐being of the region (Ives & Messerli, 1989).

### 2.2 Species occurrences

#### 2.2.1 Field survey

Occurrences of tiger were collected through field surveys from December 2019 to December 2022, primarily using foot and vehicle surveys. Direct and indirect evidences were recorded during trail transects of 1.5 km in length, laid in a 2 × 2 km grid (depending on accessibility) as per the methods of Buckland et al. (1993) and Sathyakumar et al. (2011). Indirect evidence included scats, scratches and pugmarks, while direct sightings of tiger were also recorded. A total of 381 tiger scats were identified based on secondary evidence such as diameter range and the presence of associated ancillary signs like tracks (Johnsingh, 1992; Karanth & Sunquist, 1995). In total, 502 occurrence records were collected during the field survey in Assam.

#### 2.2.2 Camera trap survey

Occurrence points of tiger were collected through camera trap surveys from December 2020 to December 2022. We used a spatial capture–recapture approach (Karanth, 1995) and deployed one pair of camera traps in the centroid of each 2 × 2 km grid, based on accessibility. The study utilized both infrared and white‐flashed Cuddeback models (H1453 and X‐change). For each site with the maximum probability of carnivore usage, GPS points were recorded using a handheld Global Positioning System receiver (Garmin Etrex_10 and 30). A total of 70 occurrence points were captured through the camera trap monitoring.

#### 2.2.3 Literature review

In addition to conducting field surveys, we gathered tiger occurrence data from various sources including published literature, reports and news articles, with a focus on camera trap locations. Our primary data extraction was based on the comprehensive tiger report published by the National Tiger Conservation Authority in India, which encompasses camera trap occurrences. Through this secondary source, we collected a total of 899 occurrence points.

For modelling tiger distribution in IEHR, only presence data (*n* = 1471) was used (Elith et al., 2006). To prevent overfitting, we retained one presence location per 5 km^2^ grid cell, removing multiple presence points (Brown, 2014). A 5 km buffer was chosen based on species ecological knowledge (Jhala et al., 2019). We performed spThin (Aiello‐Lammens et al., 2015) in R software version 4.1.3 (R Core Team, 2022). Finally, 16% (*n* = 240 of *n* = 1471) occurrence records were retained for the final model (Figure 1).

### 2.3 Environmental variables

The combination of habitat variables with climatic and anthropogenic variables has been widely acknowledged in numerous studies for producing ecologically more meaningful outcomes (Chmura et al., 2021; Gómez‐Pérez & Pardo‐González, 2021; Penjor et al., 2019). Initially, we defined 29 environmental variables across four categories: anthropogenic activities, habitats, topography and bioclim. Elevation data were obtained from the Worldclim (www.worldclim.org/), and climate data from 1970 to 2000, obtained from CHELSA (Climatologies at high resolution for the earth's land surface areas) (Karger et al., 2017) with the 1 km resolution. We considered human footprint, human modification and distance from roads under anthropogenic activities (see Appendix S1).

We used environmental data from the Community Climate System Model (CCSM5) to predict potential future distribution. The model considers an increase in globally averaged surface temperature between 1850 and 2005, which is larger than the observed increase by about 0.4°C (Gent et al., 2011). To simulate the model, we selected two Representative Carbon Pathways (RCP) greenhouse gas (GHG) emission scenarios for the 2050s (2041–2060). RCP 4.5 is a stable scenario in which GHGs will be stabilized due to green technologies, and the radiative force will extend up to 4.5 W/m^2^ by 2100 relative to pre‐industrial values (Moss et al., 2008). However, RCP 8.5 is a scenario of faster economic growth, overestimation of carbon intensity, overaggressive coal use and overpricing of renewables relative to fossil fuels (Burgess et al., 2020; Hausfather & Peters, 2020; Schwalm et al., 2020). RCP 8.5 represents the highest greenhouse gas emission scenario, corresponding to a projected increase of 2.0–3.78°C in global mean surface temperature (Van Vuuren et al., 2011).

We used data on global land use and land cover change simulations for the years 2050 from the GeoSOS global database to project future scenarios for human land use changes (Liu et al., 2017). This simulation combined MODIS land cover categories into six classes and predicted the changes from 2010 to 2100 under four scenarios of the Intergovernmental Panel on Climate Change (IPCC, 2014) Special Report on Emission Scenarios using the Future Land Use Simulation (FLUS) system. We included land use change scenarios of A1B (moderate increase in land use across all resources) in the model.

To reduce multicollinearity among the predictor variables and the overfitting of the model, we performed variance inflation factor (or VIF) in R (Graham, 2003; Naimi & Araujo, 2016). The variable with the highest VIF was removed and computed for the remaining variables until the VIF was below 10 (Hazarika et al., 2022). Furthermore, a specific set of variables was employed for the modelling process, focusing on minimizing multicollinearity and studying the local phenology of the species (Table 1).

**TABLE 1:** Environmental variables used in modelling of potential tiger habitat in the Indian East Himalayan Region.

Variables	Environmental variable unit	HLCAm	LCAm	Cm
Bio15	Precipitation Seasonality (Coefficient of Variation)	✓	✓	✓
Bio18	Precipitation of Warmest Quarter	✓	✓	✓
Bio19	Precipitation of Coldest Quarter	✓	✓	✓
Bio2	Mean Diurnal Range	✓	✓	✓
Bio3	Isothermality	✓	✓	✓
Forest type	Forest cover	✓		
NDVI	Normalized Difference Vegetation Index	✓		
Tree canopy	Tree canopy	✓		
LULC	Land cover	✓	✓	
Ele	Elevation	✓	✓	✓
Slope	Slope	✓	✓	✓
Ecu roads	Euclidian distance to roads	✓	✓	
Ecu rivers	Euclidian distance to river	✓	✓	
Human modification	Human modification	✓	✓	
Human footprint	Human footprint	✓	✓	

Abbreviations: Cm, Climate‐induced model; HLCAm: Habitat type, Land cover, Climate, and Anthropogenic, induced model; LCAm, Land cover, Climate, and Anthropogenic, induced model.

### 2.4 Species distribution modelling

The SDM ensemble approach was employed to assess the potential habitat of tigers. An ensemble of selected models based on evaluation criteria was used to improve model transferability (Guisan et al., 2017). Five different modelling techniques from the ‘sdm’ package (version 1.0‐89) in R (version 4.1.3) were initially utilized, including generalized linear model (GLM), multivariate adaptive regression splines (MARS), boosted regression trees (BRT), random forest (RF) and maximum entropy (MaxEnt). Detailed information on the SDM model, following the ODMAP protocol by Zurell et al. (2020), is provided in Appendix S3.

Pseudo‐absence selection strategies were customized for each algorithm based on recommendations (Barbet‐Massin et al., 2012). Each algorithm underwent 10 model replicates and 5‐fold cross‐validation. The occurrence data was split into 70% training and 30% test data. Presence–absence models used equal numbers of presences and pseudo‐absences, with a 5 km buffer around presence locations. The best‐fitted algorithm, based on accuracy, was selected for ensemble modelling and further analysis in ArcMap 10.8.2 and Q‐GIS 3.22.3. Model accuracy was evaluated using threshold‐independent metrics such as area under the curve (AUC), true skill statistics (TSS) and Boyce index (BI) (Boyce et al., 2002; Fielding & Bell, 1997). AUC values range from 0 to 1.0, with 0.5–0.7 considered low, 0.7–0.9 moderate and 0.9 high (Manel et al., 2001). Whereas, if scores of TSS higher than 0.5 it considered accurate at a certain detection threshold (Allouche et al., 2006). Furthermore, the null‐model approach was used for modelling validation (Raes & ter Steege, 2007) using ‘dismo’ package in r (Hijmans et al., 2021). We compared the AUC values of each model to a one‐sided 95% confidence interval derived from the null distribution of average AUC values.

### 2.5 Multiple model framework

**FIGURE 2:**
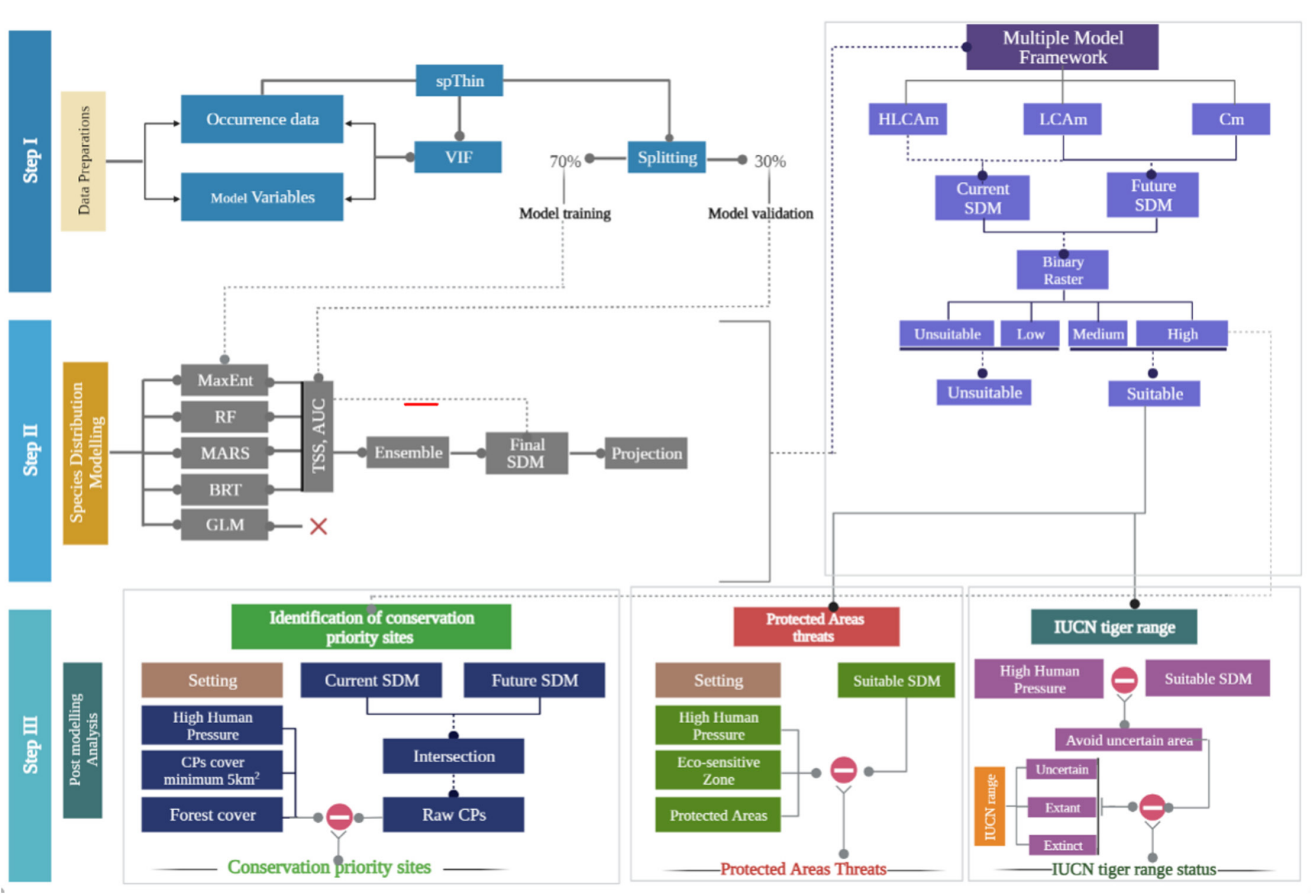
Overall framework used in the study. Multiple model framework described in the main text. Cm, Climate‐induced model; HLCAm, Habitat type, Land cover, Climate, and Anthropogenic, induced model, LCAm, Land cover, Climate, and Anthropogenic, induced model.

SDMs have played a crucial role in recent decades in understanding the potential habitat of plants and animals and in creating conservation plans for threatened species. While most SDMs employ a single model and relevant variables for the target species, a multiple model approach is rarely used in research. To address this gap, we have developed a framework that utilizes two or more models to better understand their importance in a single study. The term ‘multiple’ refers to the composite of two or more models used to compare outputs and select the best conservation priority site for any given species (see detailed framework in Figure 2). This framework also helps to evaluate the uncertainty in the model and its predictive results.

#### 2.5.1 Habitat type, land cover, climate and anthropogenic induced model (HLCAm)

The baseline model (HLCAm) encompassed all essential variables for tiger distribution, excluding prey distribution. The model integrated climate, habitat type, land cover, anthropogenic factors and physiographical variables. Physiographic variables were included in all three independent models due to their significance for tigers in the IEHR. Elevation and slope were incorporated to account for the mosaic landscape of the IEHR, which comprises mountains, hills, plains and rivers. Future climate scenarios were not considered in the model, as we focused on habitat variables (NDVI, forest cover and tree canopy) that might undergo substantial changes in the near future.

#### 2.5.2 Land cover, Climate and Anthropogenic‐induced model (LCAm)

Incorporating land use in distribution models, we can enhance the accuracy of species predictions. In our study, we considered both the 2050 land use scenario and climate data. However, due to the lack of reliable future human activity data, we assumed that human activity would remain constant from the present to 2050. To address this limitation, we utilized current human footprint data for our future projections (Sun et al., 2021).

#### 2.5.3 Climate‐induced model (Cm)

Climate change is expected to have a major impact on tropical forests worldwide (IPCC, 2014), as well as on the IEHR (Deka et al., 2022). In order to assess the impact of climate change on tiger habitat, we have incorporated the Cm model into our framework. In a previous study, it was found that climate change could affect tiger habitats by 2050 and 2070 in the Sundarban region of Bangladesh (Mukul et al., 2019). Therefore, we have focused on the climatic variables that influence tiger distribution in our study. However, we recognize that our projected area may be biased and that the inclusion of land use variables could change the results (Di Febbraro et al., 2019). To address this, we have eliminated areas with high human pressure from our conservation priority analysis.

### 2.6 Post modelling analysis

The multiple model framework outputs were converted to binary maps and classified based on prediction equivalence threshold: not suitable (<0.2), low suitable (0.2–0.4), moderate suitable (0.4–0.6) and high suitability (>0.6). An ensemble of human disturbance layers, including human footprint and human modification, was classified into high disturbance (>0.3) and low disturbance (<0.3) layers. We then eliminated potential areas under high disturbance to find the projecting areas for conservation planning. Finally, suitable raster layers were converted to points and analysed using the intersect tool in ArcGIS. All analyses were conducted using ArcMap version 10.8.2, Q‐GIS version 3.22.3., R version 4.1.3. and MS Excel 2019.

#### 2.6.1 Identification of conservation priority sites

To determine conservation priorities, we focused on high and medium suitability classes from the binary reclass maps, excluding cells in the high human modification/footprint category. We selected only cells found suitable in current and future climate conditions and designated areas that are expected to remain intact in HLCAm, LCAm and Cm models. We ranked the suitability of each model under current and future climate conditions. The high suitable area under HLCAm was ranked as a very high priority, followed by medium suitability as a high priority. The suitable areas under the current LCAm and Cm were merged with HLCAm using intersect tools. For future climate scenarios, we used the same RCP 4.5 and RCP 8.5 for the 2050s, collectively ranked as a possible high priority for future climate and land use changes.

#### 2.6.2 Status of protected areas threats

To understand the threats faced by PAs, we considered the eco‐sensitive zone (ESZ) of each PA, which varies from state to state in India. To do this, we used a 1 km buffer around each PA, as recommended by the government (https://pib.gov.in/PressReleseDetail.aspx?PRID=1842610). We then used anthropogenic layers to assess the threats to each PA, including human footprints, human modifications and distance from roads. We excluded datasets that are uncertain or not yet accessible, such as conflict and cattle data. After selecting the relevant layers, we merged them for further analysis and extracted a binary map from the ESZ of each PA. Finally, we calculated the threats to PAs in the form of anthropogenic pressure within the ESZ boundary.

#### 2.6.3 Distribution status under IUCN tiger range

The IUCN range of particular species counts as an authentic source of data. If the species is endangered, then their information is more significant. Here, we evaluated the IUCN tiger range with our predictive model for the IEHR. To do this, we downloaded the tiger range polygon from the IUCN red list (Goodrich et al., 2022) and accessed the suitability under three ranges (extant, presence uncertain, and extinct). Here, we excluded the high human modification and high human footprint layers for more accuracy in the distribution area. Finally, we improved the IUCN range map according to our study models and true presence.

## 3 RESULTS

### 3.1 Model performance and important variables

Among the five predictive algorithms, GLM was omitted from the final output for three models due to low predictive accuracy (AUC below 0.8). Our models achieved AUC values above 0.8 and TSS values above 0.5 (Elith et al., 2006) (Table 2). Additionally, the null model confirmed that the three SDMs (HLCAm, LCAm, and Cm) outperformed a random model significantly, with average AUC values of 0.731, 0.749, and 0.692, respectively. The ensemble forecast outputs (weighted mean) for the three independent models showed a positive average BI value of 0.951. The BI values indicated that the predictive maps under HLCAm (BI = 0.977) and LCAm (BI = 0.975) have high accuracy in suitable areas, while the accuracy of the predictive map under Cm (BI = 0.902) is comparatively lower than that of HLCAm and LCAm (Table 2).

**TABLE 2:** Model performance metrics obtained from fivefold cross‐validation: area under the curve (AUC), true skill statistics (TSS), and Boyce Index (BI).

Methods	AUC			TSS			BI		
	HLCAm	LCAm	Cm	HLCAm	LCAm	Cm	HLCAm	LCAm	Cm
RF	0.88	0.88	0.83	0.66	0.66	0.66	0.991	0.993	0.903
Maxent	0.82	0.82	0.85	0.56	0.52	0.51	0.984	0.974	0.898
Mars	0.81	0.85	0.81	0.54	0.52	0.50	0.961	0.971	0.911
BRT	0.81	0.81	0.80	0.54	0.55	0.54	0.972	0.962	0.899
GLM	0.72	0.72	0.66	0.39	0.32	0.36	0.815	0.805	0.855

Abbreviations: Cm, Climate‐induced model; HLCAm, Habitat type, Land cover, Climate, and Anthropogenic, induced model; LCAm, Land cover, Climate, and Anthropogenic, induced model.

The variable importance analysis in the multiple model framework has indicated that certain predictive variables are consistently important across multiple models. However, uncertainties may arise when attempting to identify potential distributions in some cases. For instance, under HLCAm, the three most important variables in predicting the distribution of tigers are elevation, human modification, and bio14. In the case of LCAm and Cm, the three most important variables are elevation, bio14, and bio19. Although each model has different predictive maps and accuracy, the overall variable contributions remain consistent. Elevation is particularly crucial in the distribution of tigers in IEHR (see Appendix S2). Furthermore, marginal response curves have been generated to demonstrate how the predicted probability of presence changes when each environmental variable is varied while holding all other variables constant (see Appendix S2).

### 3.2 Current projected distribution of Bengal Tiger in IEHR

**FIGURE 3:**
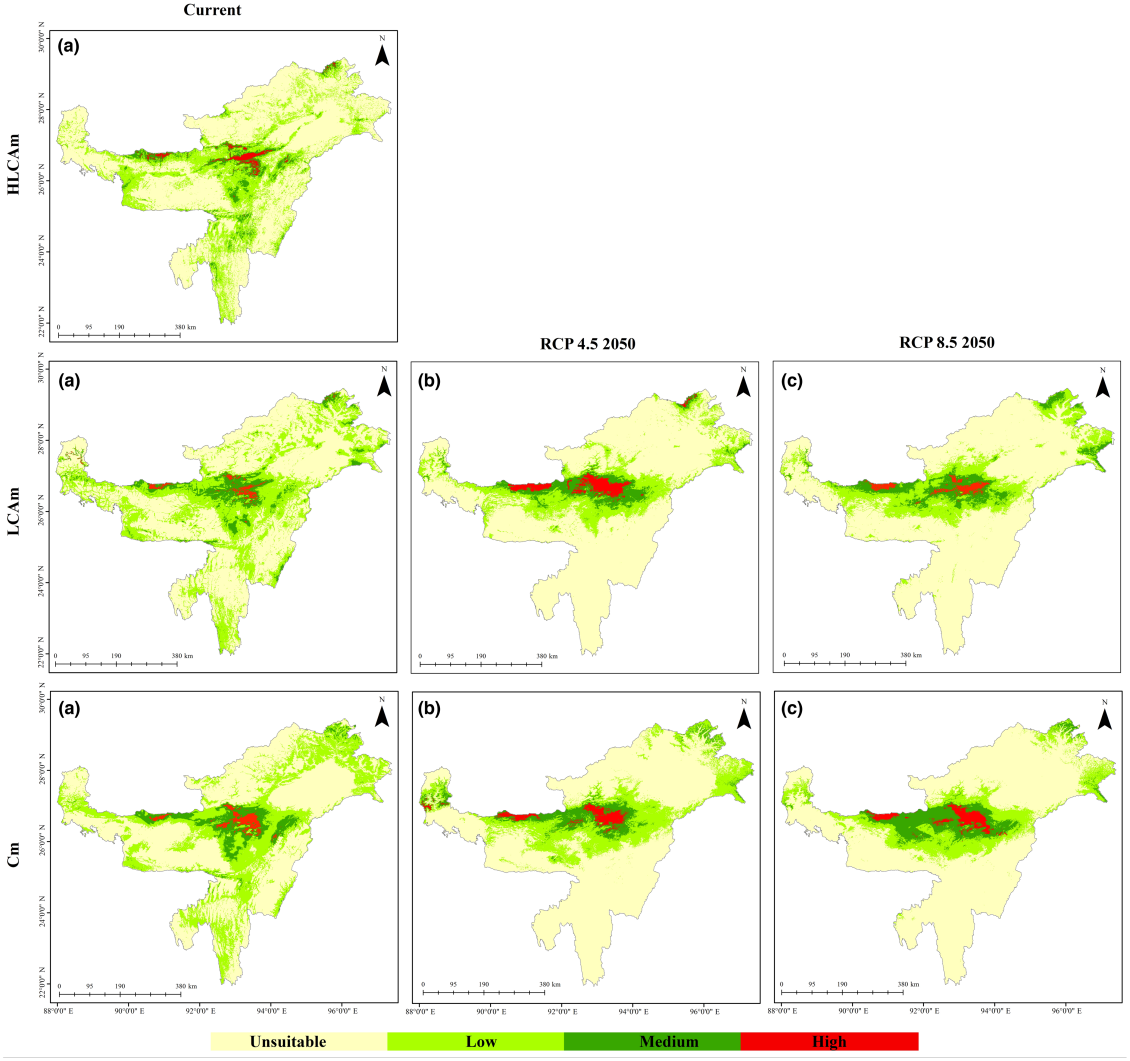
Distribution of tiger in the Indian East Himalayan Region. (a) Current potential habitat under three models HLCAm, LCAm and Cm, (b) Future potential habitat under climate scenario of RCP 4.52050 for LCAm, and Cm, (c) Future potential habitat under climate scenario of RCP 8.52050 for LCAm and Cm.

**FIGURE 4:**
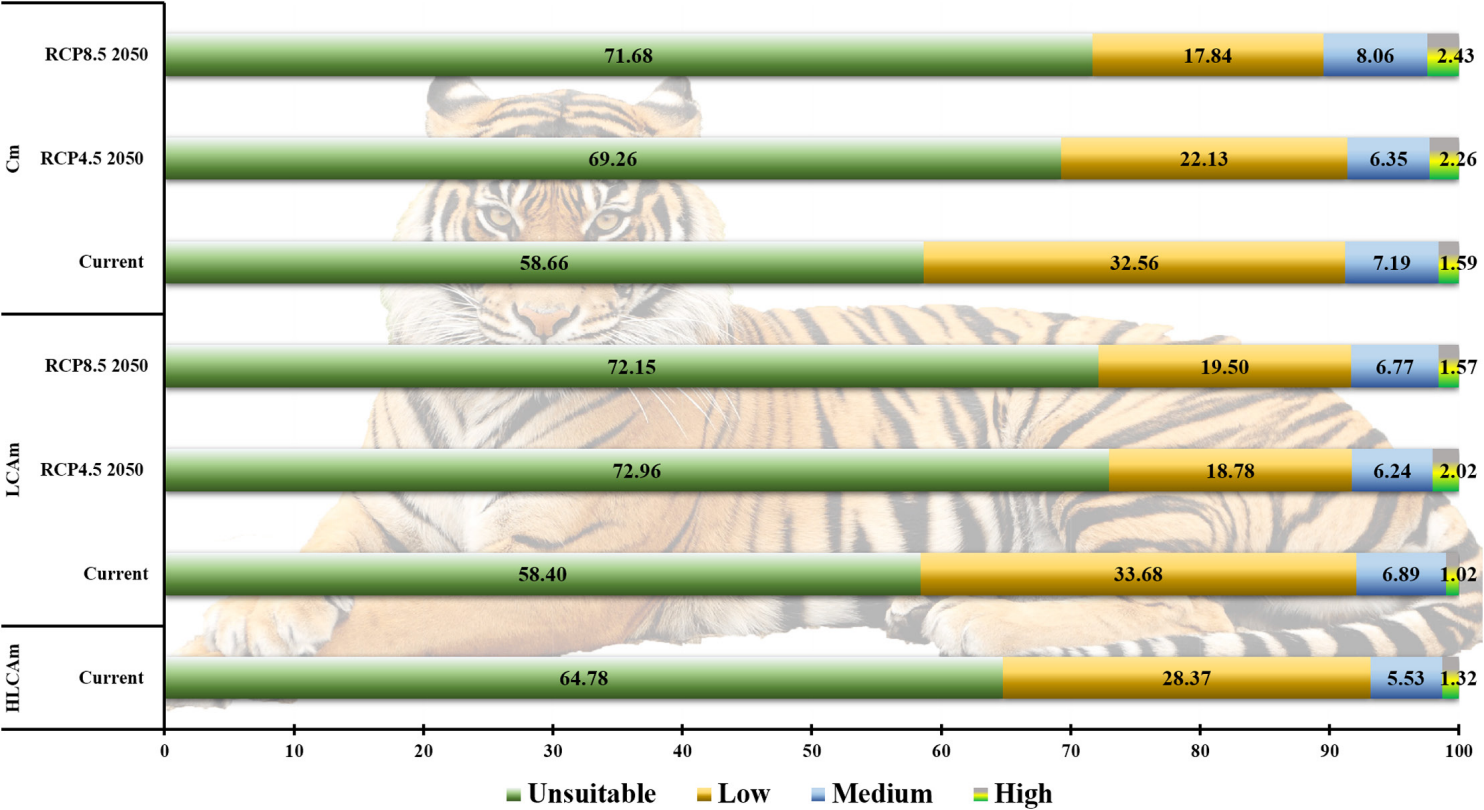
Percentage of distributional status of tiger in the Indian East Himalayan Region under current and future potential habitat under three models HLCAm, LCAm and Cm.

In the present study, Brahmaputra Valley and Karbi‐Anglong hills in the IEHR were found as strongholds of tiger habitat (see Figure 3). According to the HLCAm model, tigers have 6.85% of suitable habitat (1.32% high, 5.53% medium), solely in the Brahmaputra valley (Table 3). The LCAm model projects the current suitable areas to be 7.91% (1.02% high, 6.89% medium) (see Figure 4 and Table 3). As we removed habitat variables from the model, the rate of suitable areas increased, indicating that including habitat type variables results in more accurate estimations. However, using habitat variables for future projections may introduce biases. By comparing the HLCAm and LCAm models, we found that the Cm model projected the highest suitable area, covering 8.78% (1.59% high, 7.19% medium) (see Figure 4 and Table 3). This is because land cover, habitat, and anthropogenic variables were not considered in the model. We can evaluate the importance of different sets of variables necessary for a specific species. Finally, our study highlights that climate, habitat, and land cover variables are crucial aspects of any SDM study.

**TABLE 3:** Potential tiger habitat in response to climate change in Indian East Himalayan Region.

Class	HLCAm	LCAm			Cm		
	Current (km^2^)	Current (km^2^)	RCP4.5 2050 (km^2^)	RCP8.5 2050 (km^2^)	Current (km^2^)	RCP4.5 2050 (km^2^)	RCP8.5 2050 (km^2^)
Unsuitable	177171.14	159727.93	199526.55	197334.40	160419.40	189421.61	196037.60
Low	77581.32	92110.09	51373.44	53333.26	89052.61	60523.75	48781.41
Medium	15133.31	18850.65	17057.57	18520.33	19664.61	17374.46	22032.99
High	3605.13	2802.23	5533.34	4302.13	4353.49	6170.28	6638.11

**FIGURE 5:**
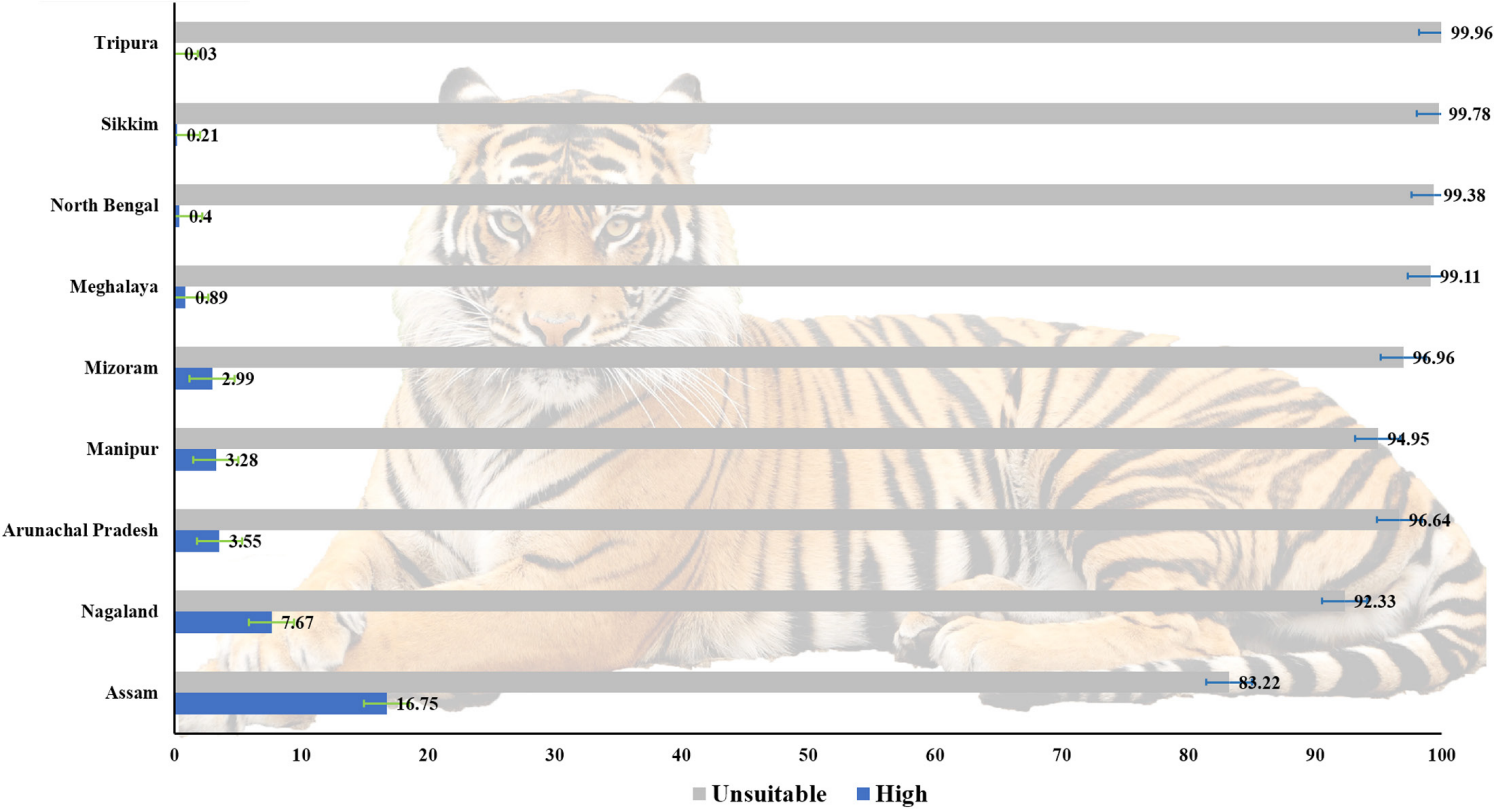
State‐wise suitability and unsuitability status of tiger in the Indian East Himalayan Region.

Our analysis revealed that Assam state, has the highest potential distribution of tigers in the East Himalayan region. State‐wise analysis indicated that 16.75% of suitable habitats were found in Assam, with the remaining suitable habitats distributed across Nagaland, Arunachal Pradesh, Manipur, Mizoram, Meghalaya, North Bengal, Sikkim and Tripura (Figure 5, Table 4). Consistent with the findings, Assam alone reported 200 tigers in 2021, according to three of its tiger reserves, namely Kaziranga, Manas and Orang. Moreover, our analysis of protected area habitat showed that the Kaziranga Tiger Reserve in Assam contributes the most to potential tiger habitat.

**TABLE 4:** State‐wise evaluation of potential tiger habitat in response to climate change and land use change in the Indian East Himalayan Region.

States	Suitability	HLCAm	LCAm			Cm		
		Current (%)	Current (%)	RCP4.5 2050 (%)	RCP8.5 2050 (%)	Current (%)	RCP4.5 2050 (%)	RCP8.5 2050 (%)
Assam	Unsuitable	83.22	80.67	73.19	75.74	75.75	76.14	66.09
	Suitable	16.75	19.33	26.81	24.26	24.25	23.86	33.91
Arunachal Pradesh	Unsuitable	96.64	95.86	95.76	94.30	97.93	94.88	96.16
	Suitable	3.55	4.05	4.15	5.62	2.07	5.12	3.84
Nagaland	Unsuitable	92.33	95.10	99.92	95.74	86.16	99.39	90.9
	Suitable	7.67	4.90	0.08	4.26	13.84	0.61	9.1
Manipur	Unsuitable	94.95	98.31	100.00	100.00	99.23	100.00	100.00
	Suitable	3.28	1.69	0.00	0.00	0.77	0.00	0.00
Mizoram	Unsuitable	96.96	99.52	100.00	100.00	100.00	100.00	100.00
	Suitable	2.99	0.48	0.00	0.00	0.00	0.00	0.00
Tripura	Unsuitable	99.96	99.91	100.00	100.00	99.99	100.00	100
	Suitable	0.03	0.09	0.00	0.00	0.01	0.00	0.00
Meghalaya	Unsuitable	99.11	97.43	99.85	99.87	99.13	99.45	98.9
	Suitable	0.89	2.57	0.15	0.13	0.87	0.55	1.10
North Bengal	Unsuitable	99.38	96.65	98.89	97.26	99.64	92.87	98.77
	Suitable	0.40	3.35	1.11	2.74	0.36	7.13	1.23
Sikkim	Unsuitable	99.78	92.63	95.66	96.30	99.73	84.70	97.27
	Suitable	0.21	7.37	4.34	3.70	0.27	15.30	2.73

### 3.3 Distribution under future climate change and land use scenarios

In the future, the IEHR is projected to have an increased potential for tiger habitats. However, according to the LCAm model, there will be a decline in tiger habitat due to human‐induced land use changes and climate change by the 2050s under the RCP 8.5 scenario. Despite this, the Cm model indicates that the IEHR could still maintain significant tiger habitats, accounting for 2.26% and 2.43% of the potential habitat under RCP4.5 and RCP8.5, respectively, by the 2050s. However, these habitats are expected to decrease due to ongoing land use changes and anthropogenic activities (Table 3).

Regarding the specific regions within the IEHR, high‐altitude states like Arunachal Pradesh and Sikkim are projected to retain tiger habitat under the RCP4.5 scenario by 2050, but this habitat will decline under the more severe RCP8.5 climate scenario. The Assam‐Bhutan border is expected to have the most potential tiger habitat by 2050 under RCP4.5, but this distribution is anticipated to gradually decrease under RCP8.5 by 2050. Overall, Assam, along with the border areas of Bhutan, Nagaland, Meghalaya and Arunachal Pradesh, are likely to continue hosting tiger habitats in the future.

### 3.4 Identification of priority conservation sites

**FIGURE 6:**
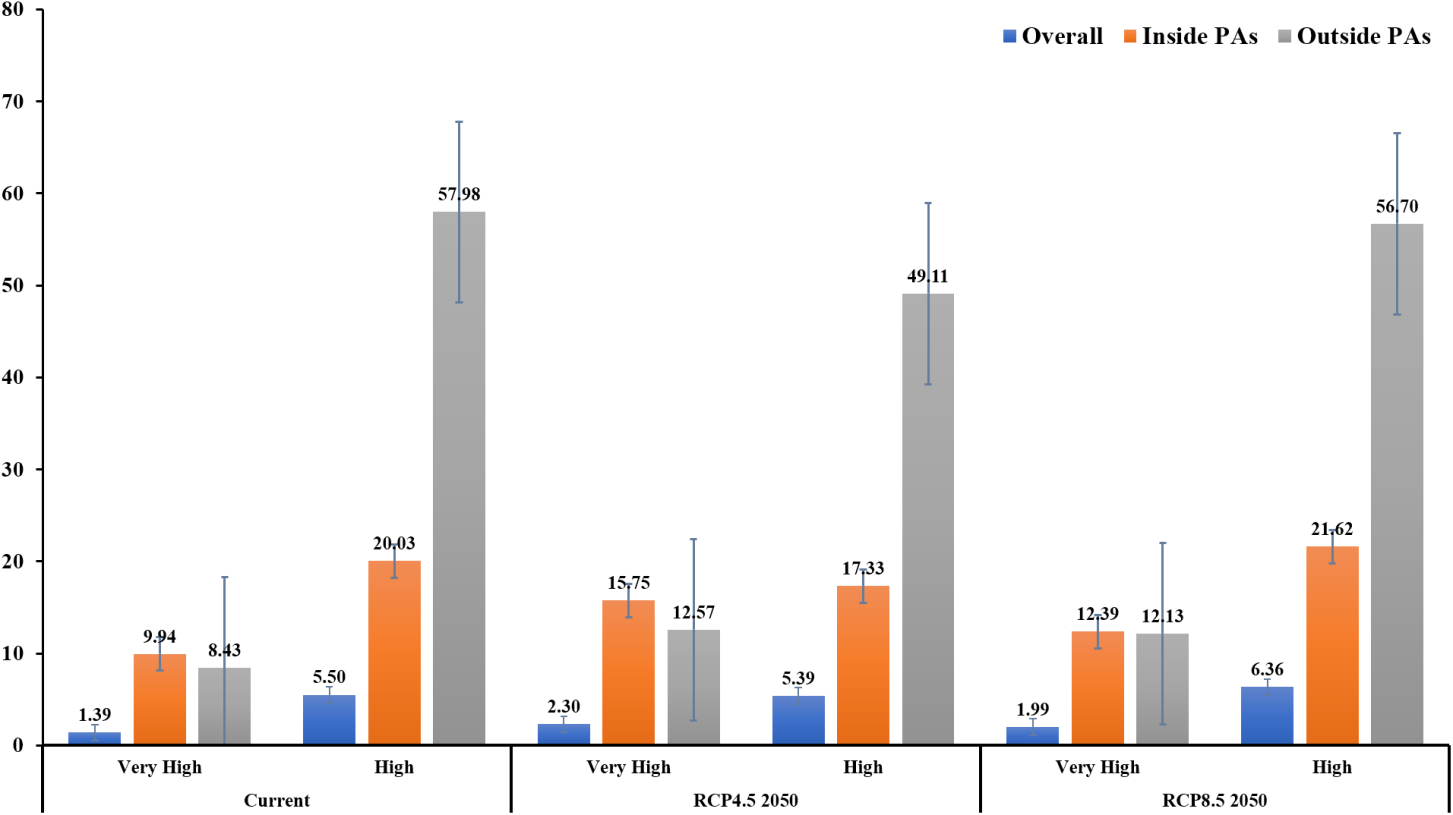
Status of Conservation priority sites (Overall, inside protected areas and outside protected areas) in the Indian East Himalayan Region.

To achieve the conservation objective, it is crucial to protect tigers in situ, known as conservation priority areas (CPs), within the IEHR. In order to identify these areas, we excluded 52,582.52 km^2^ area with high human density from the total IEHR. We then considered 220,908.38 km^2^ of areas with low human density to determine the priority areas. Under the current climate conditions, the average conservation priority areas (HLCAm, LCAm and Cm) cover 3.40% of the total area, with the majority of these areas confined in riverine corridors and the Kaziranga Tiger Reserve and Karbi Hills (Figure 10). Interestingly, the conservation priority areas found outside PAs are higher than those inside PAs. In fact, 8.42% of the highest conservation priority areas are located outside the PAs, and 57.97% of the high‐potential area is also outside them (Figure 6). These areas outside of PAs also require protection for the survival of the species, with the Karbi Hills and Naga Hills being of utmost priority.

### 3.5 Threats to protected areas

**FIGURE 7:**
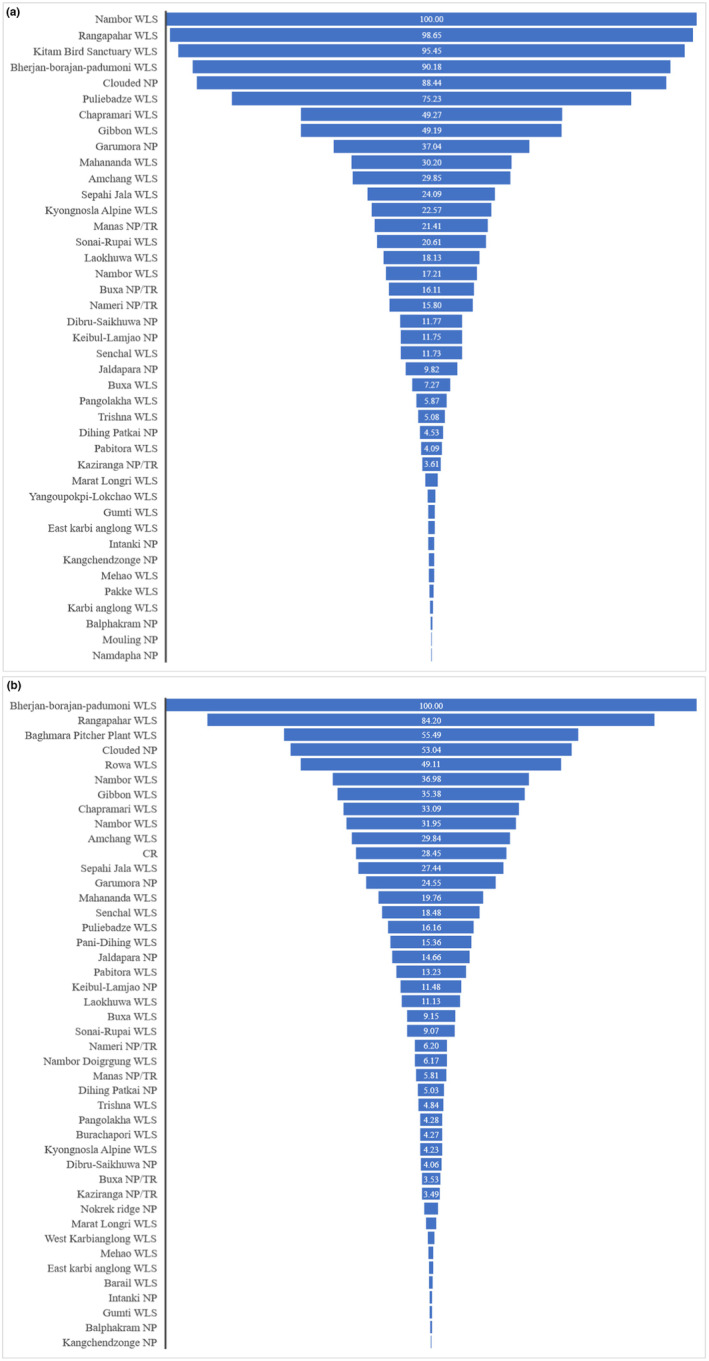
Protected areas (PAs) threats by high human pressure within eco‐sensitive zone (a) inside PAs threats and (b) outside PAs threats.

**FIGURE 8:**
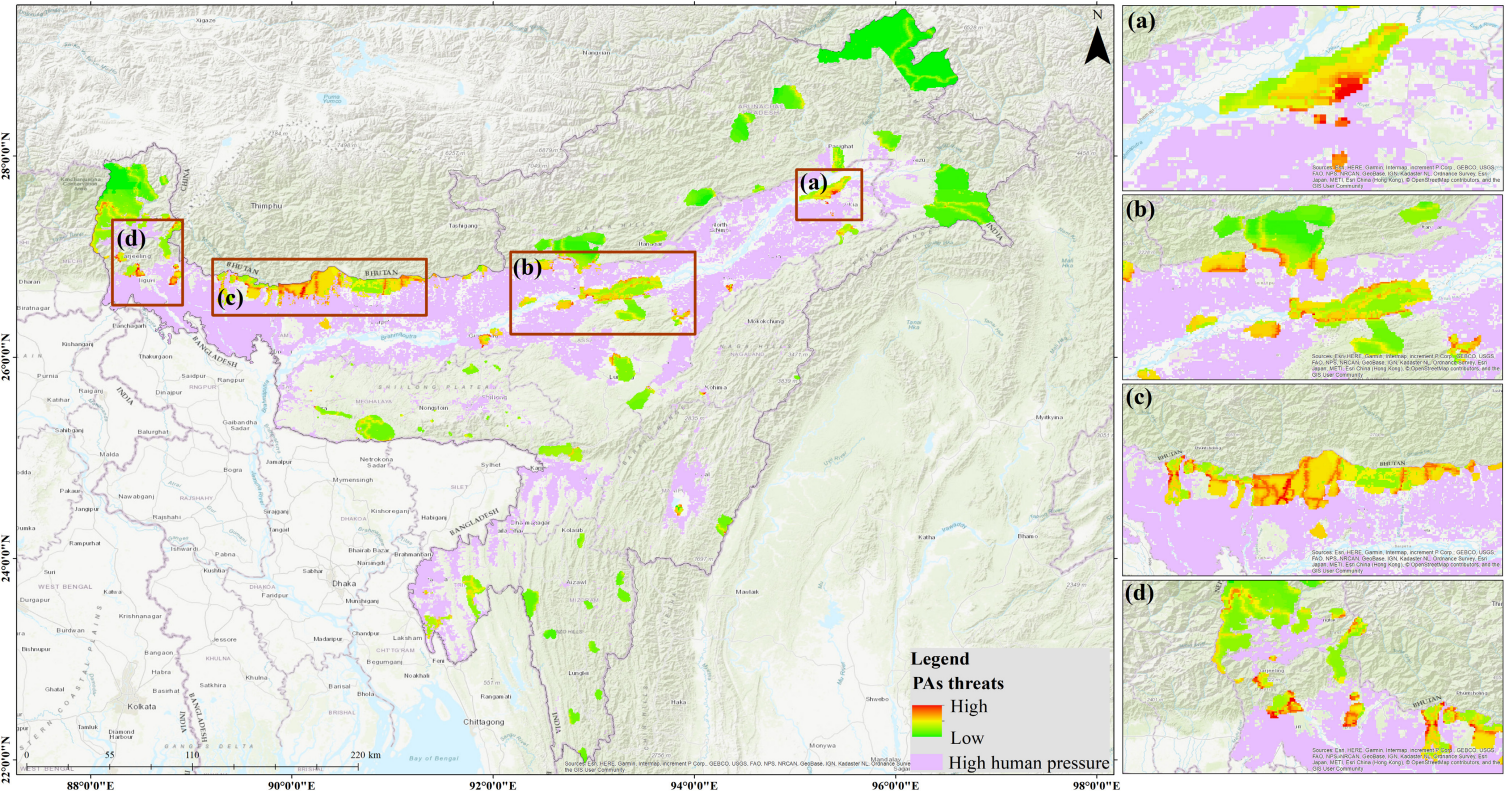
Distribution of protected areas (PAs) threats within Eco‐sensitive zone in the Indian East Himalayan Region. (a) Eastern Assam including Dibru‐saikhuwa National Park, (b) Central Assam including Kaziranga National Park and Tiger reserve, Nameri National Park and Tiger reserve landscape, (c) Manas‐Buxa landscape and (d) North Bengal and Sikkim landscape.

To understand the threat to PAs in the IEHR, we analysed human pressure and found that Assam had the highest threat level among all the states, both inside and outside PAs, with 41.67% and 45.95%, respectively. Assam alone accounted for 65.43% of the high human pressure and causing threat to tiger population. The LCAm and Cm scenarios predict an increase in PAs threats. The IEH holds 101 PAs (http://www.wiienvis.nic.in/Database/Protected_Area_854.aspx) that help maintain diversity of flora and fauna. However, we showed that 44 PAs have high threats within the eco‐sensitive zone and 41 PAs have threat inside the periphery. Wildlife sanctuaries (WLSs) are facing severe threats than National parks (NPs) in the IEHR, with 27 WLSs and 24 NPs facing threats either inside or outside PAs (Figure 7a,b). This is primarily due to the lack of management issues and manpower with concerned department. The Sonai‐Rupai WLS, Laukhuwa‐Burachapori WLS, East Karbi‐Anglong Hills, Nameri NP, Kaziranga NP, Groumara NP and Buxa NP are among the major PAs crucial for tiger habitat (Figure 8).

### 3.6 Distribution status under IUCN tiger range

**FIGURE 9:**
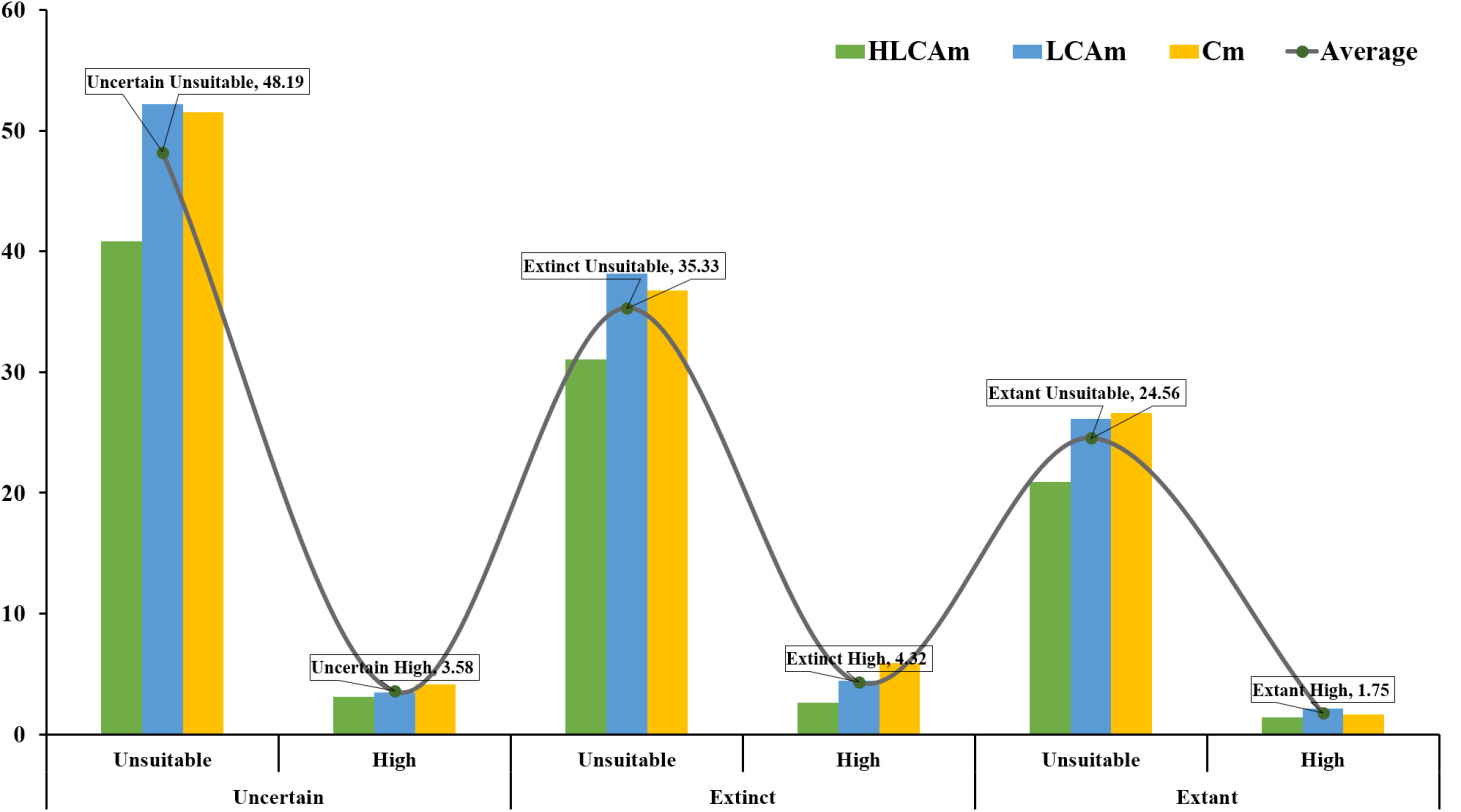
Evaluation of the designated IUCN tiger range with the species distribution models.

We estimated the present suitable and unsuitable areas within the three IUCN distribution classes: uncertain, extant and extinct. Our analysis revealed that even under the extinct range, the tiger could still hold 4.32% of high suitable habitat, as confirmed by our field survey data. Our SDM predictive map also showed valid tiger presences covering 0.89% of high suitable area under the uncertain range (Figure 9). However, the PAs designated as tiger reserves are not currently considered extant suitable habitats. Our evaluation indicates that the IEHR currently has 30% of the tiger's current residential area according to the IUCN range, but this area can increase after re‐evaluation.

## 4 DISCUSSION

Large carnivores require extensive territories, making them vulnerable to habitat fragmentation and isolation (Thatte et al., 2017). Similarly, climate change and human‐induced land cover change have altered the habitat range of large carnivores, including tigers (Mukul et al., 2019). Through this study, we assessed conservation priority areas and their possible range shift due to climate and land cover scenarios. We found that a large potential area in the Brahmaputra valley could be lost in the future, whereas suitable areas could shift eastward or northward. These projected priority sites require proactive conservation planning, including establishing safe corridors, expanding protected areas or mitigating human–carnivore conflict (Pratzer et al., 2023). Our study has created a protected areas threat assessment that can guide conservation planners and provide reliable output for immediate conservation planning.

### 4.1 Variable importance of Bengal tiger in IEHR

The factors influencing tiger habitat suitability are intricate and interconnected. In our study, we identified temperature, precipitation, land cover, anthropogenic variables, elevation and slope as significant contributors to tiger distribution. Among these factors, precipitation‐related variables exhibited a greater influence than temperature across all three models employed. Tigers prefers areas with moderate rainfall, as excessive or insufficient precipitation can hinder their ability to locate prey. We found that the majority of potential tiger habitat in IEHR falls within the range of 500–1500 mm. Our study underscores the importance of precipitation during the wettest month, as well as the coldest and warmest quarters, for tiger habitat. In contrast, Rather et al. (2020) emphasize the significance of precipitation during the driest months and quarter for the Bandhavgarh Tiger Reserve in India. These variations in precipitation variables are primarily influenced by the study area's location and the local phenology of tiger habitat. Although the importance of these variables may differ, precipitation remains the key determinant for tiger habitat.

Elevation was found to be a crucial factor in tiger distribution in both the LCAm and Cm states, consistent with the study by Bajaj and Amali (2019), which highlighted the limiting effect of altitude on tiger populations, favouring lower elevation areas (Jhala et al., 2008). Additionally, Seidensticker and McDougal (1993) observed that tigers prefer areas with undulating slopes and abundant water. Our results indicate a high probability of tiger presence on slopes of up to 20 degrees. Gentle slopes, preferred by most grazing animals (Schaller, 1967), are also favoured by tigers (Karanth & Nichols, 1998). Furthermore, Carter et al. (2013) found that tigers highly prefer land cover patches with more grassland. Taking these factors into account, we observed that the majority of tiger habitats are located within the intricate landscape of grassland and woodland, such as in Kaziranga and Orang of IEHR. Additionally, tiger habitats are frequently found in proximity to rivers or streams, connecting lowland forests or wooded grasslands (Chandran et al., 2012). Our findings also revealed that a significant number of habitats were located near rivers, which aligns with the findings of Imam et al. (2009) stating that areas in proximity to water sources are more suitable for tiger habitat. Furthermore, Duangchatrasiri et al. (2019) also suspect that low‐slope forests near streams are also preferred habitats of major tiger prey base (Jhala et al., 2019) thus these areas are crucial for tiger site use.

Our baseline model (HLCAm) identified forest type, tree canopy extent, and NDVI (Normalized Difference Vegetation Index) as significant factors in tiger distribution. Kanagaraj et al. (2011) also emphasized the importance of habitat variables in achieving specific outcomes. Moderate dense forest with canopy cover above 50% strongly supports tiger habitat, consistent with previous studies (Sarkar et al., 2017; Sinha et al., 2012). Our field observations in Assam prove that compact bamboo forests in complex terrain and riverine forests are preferred by tigers (Sarkar et al., 2017) while heavily degraded forests also attract in certain landscapes, that is grassland with degraded forest (Pudyatmoko et al., 2023). Habitat sharing among tigers has been observed in Chandoli National Park's evergreen forest, reflecting patterns in the IEHR of Dihing‐Patkai National Park (Imam et al., 2009). Furthermore, the humid subtropical climate of the region supports mixed moist deciduous forests dominated by trees like *Shorea robusta* and *Lagerstroemia* sp. Our field observations in certain areas of Assam, characterized by a combination of mixed forests and tall grasslands, align with these findings and further validate our model. From a management perspective, this emphasizes the importance of safeguarding extensive natural ecosystems with minimal human impact to accommodate species that are sensitive to human disturbance and have large home range requirements (Penjor et al., 2021, 2020b).

In addition to natural factors, the habitat of tigers is heavily influenced by human disturbances. Our findings indicate that human modifications have a significant impact on tiger habitat in the IEHR, followed by the distance from roads and human footprint. Potential tiger habitats are found within 2 km from human‐modified areas, which raises concerns about the potential for human–tiger conflicts and the loss of habitat due to human activities (Warrier et al., 2020). Furthermore, our study found no potential habitats within a minimum distance of 3 km from roads, emphasizing the challenges faced by tigers in areas close to road networks and the associated human footprint (Karwariya et al., 2017; Prajapati et al., 2014). This highlights the need to address the threats posed by human modifications and road construction to ensure the conservation of tiger populations and their habitats (Carter et al., 2014; Oishimaya, 2020). Proactive conservation planning, including the expansion of protected areas and measures to mitigate human–carnivore conflicts, is crucial in this regard.

### 4.2 Current distribution of Bengal tiger in IEHR

The HLCAm and LCAm models indicate proximity to existing protected areas, suggesting the potential for safe corridors to enhance the tiger population along the Assam‐Meghalaya and Assam‐Nagaland borders. These models align with the current distribution of tiger ranges (Goodrich et al., 2022). While the climate‐induced model (Cm) focuses on climate‐suitable areas while avoiding human habitat, revealing that tigers are primarily influenced by land cover rather than climate change. However, caution is needed as SDM alone may overestimate the tiger's geographic distribution, as noted in previous studies (Tian et al., 2014). Our study demonstrates the value of employing three independent SDM models to delineate the species' habitat and inform conservation planning. The projected suitable tiger locations in the IEHR vary based on different protected area landscapes that support prey populations. Urgent attention is required for conservation planning in suitable locations outside protected areas.

Assam in the IEHR offers the highest potential tiger habitat with abundant prey and diverse habitat in the Brahmaputra floodplains (Jhala et al., 2015). However, densely populated areas hinder tiger movement, limiting their habitat. Protected areas in Meghalaya, particularly Nongkhyllem WLS, show potential for tiger presence supported by prey populations (Jhala et al., 2019; Kumar & Marcot, 2010). In Nagaland, suitable potential habitats exist, but no tigers have been spotted since 2016 (Nijhawan, 2016). The northeastern part of Nagaland, connecting Shangphan WLS with Arunachal Pradesh and Assam, may have tiger presence, but intense poaching activity and limited prey base raise concerns. Assessing prey populations in projected suitable areas is crucial for safeguarding tiger habitat. Ongoing efforts are needed to protect and conserve tiger habitats in the IEHR. Close monitoring of the tiger population in Assam and potential habitats in Meghalaya and Nagaland is vital to prevent further decline due to human activities. Preserving prey populations and enforcing anti‐poaching laws are essential for ensuring the survival of tigers in the region.

Our study model indicates that the southern region of the IEHR has limited tiger habitat. Despite having good forest cover, this area lacks suitable habitat for tigers. We identified several factors contributing to this limitation, including high precipitation, dense evergreen forests, rugged terrain, and steep slopes, which make it challenging for tigers to navigate and establish territories (Singh et al., 2009). Additionally, there is a lack of comprehensive data on the prey base for tigers in this region (Jhala et al., 2019). However, it is worth noting that tigers have been sighted in areas with lower suitability, such as Manipur (Samom, 2019). In 2021, a tiger sighting was recorded in the Dampa Tiger Reserve in Mizoram, after a 7‐year gap, which aligns with our projected suitable areas. Despite these sporadic sightings, our study also reveals that many previously reported tiger sightings are no longer feasible in the current landscape scenario. For instance, the Gumti Wildlife Sanctuary in Tripura, which was once known to have tigers, no longer supports their presence (Gupta, 1988). Our model suggests that Tripura has lost its ability to host viable tiger habitats due to factors such as high human pressure and habitat fragmentation. The migration and intermixing of populations in different fragments have been adversely affected following the loss of connecting forest corridors between fragments resulting from non‐traditional jhooming (Gupta, 2000).

Furthermore, there have been recent sightings of tigers in high‐altitude areas of Arunachal Pradesh, Sikkim, and North Bengal, which have expanded our knowledge of the species' range (Global Tiger Forum, 2019). This indicates the presence of potential tiger habitat in our study model. In the Dibang Valley District of Arunachal Pradesh, a tiger was photographed at 3630 metres above sea level, confirming its ideal habitat (Adhikarimayum & Gopi, 2018). Our model also suggests that the Dibang Valley Wildlife Sanctuary and areas outside this protected area in Dibang Valley District holds a high potential for tiger occurrence. Additionally, photographic evidence confirms the presence of tigers in North Sikkim, although there is limited information available regarding their status and distribution (WWF, 2019). Our findings indicate that Pangolakha Wildlife Sanctuary and Fambonglho Wildlife Sanctuary in Sikkim are likely to host a significant portion of potential tiger habitat (Umariya et al., 2021). Specifically, the Himalayan mixed deciduous forests are a prominent habitat for tigers, and recent photographic evidence in the Neora Valley National Park and the Buxa Tiger Reserve further support the presence of tigers in these areas (Chatterjee et al., 2020; Guha, 2022). These findings suggest that tiger populations in the IEHR may be more widespread than previously believed. Overall, our projections of suitable areas for tigers are well‐informed and can be used to guide conservation decision‐making. However, continued efforts are necessary to protect and conserve these habitats and prevent further decline due to human activities. The expansion of tiger ranges into high‐altitude areas and the rediscovery of tiger populations in previously unrecorded areas highlight the need for further research and conservation efforts in the region. Additionally, it is essential to implement measures to reduce human–wildlife conflict and address the drivers of habitat loss, such as poaching and land use change, to ensure the survival of these magnificent animals in the IEHR under future land use and climate change scenarios.

### 4.3 Impact of land use and climate change in the future

Our research revealed that tiger habitats in the IEHR are facing imminent threats arising from two main sources, human‐induced land cover changes (Penjor, Kaszta, et al., 2021; Penjor, Wangdi, et al., 2021) and the impact of climate change (Mukul et al., 2019; Rather et al., 2020). The ever‐increasing global population has triggered substantial transformations in land use worldwide (Lambin et al., 2001), particularly within Asia (Zhao et al., 2006) and, more specifically, in the Himalayan region (Tiwari, 2000). These alterations, primarily in the form of deforestation, have proven to have a more profound impact on mammal distributions within the Eastern Himalayas than even the effects of climate change (Trisurat et al., 2015). However, climate change does exert a significant influence on mammal species, resulting in behavioural shifts, loss of suitable habitats, range expansions, and/or shifts (Gossmann et al., 2019). It is worth mentioning that different species may be impacted differently by the combined effects of land use and climate change. For instance, a study focusing on the Himalayan brown bear highlights that their habitat is more threatened by climate change than human land use (Dar et al., 2021). Conversely, our study specifically examining tigers has shown that they are more susceptible to the consequences of human‐induced land cover changes. In light of our comprehensive analysis, it becomes clear that population growth and climate change both play pivotal roles as drivers of environmental change in the Himalayan region.

Climate change has already had significant impacts on tiger habitats, as documented by several studies (Devi et al., 2018; Loucks et al., 2009; Mukul et al., 2019, 2018; Rahim et al., 2015; Rather et al., 2020; Tian et al., 2014). These impacts encompass changes in diet, with projections indicating a decline in the availability of preferred prey (Hebblewhite et al., 2014; Qi et al., 2020). Furthermore, as rising sea levels continue to destroy mangrove habitats, some tigers may be forced to shift their ranges or increase their movement in order to establish new home ranges (Mukul et al., 2019).

Our research findings indicate that the northern states of the IEHR, namely Sikkim, Arunachal Pradesh, and the hilly regions of Assam, will witness an increase in suitable habitats due to future changes in land cover and climate scenarios. By 2050, under the RCP 4.5 climate change scenario, there will be a shift in the range of suitable habitats towards the east or northward. This positive trend extends to potential tiger habitats, as similar results project an expansion northward under various climate change scenarios in the Russian Far East and northeastern China (Tian et al., 2014). However, it is concerning to note that the loss of suitable habitat for tigers has been observed under the more severe RCP 8.5 scenario, as highlighted by modelling studies (Deb et al., 2019; Mukul et al., 2019; Rather et al., 2020). Additionally, our research specifically highlights and supports that the habitat suitability in Assam is projected to decrease under the worst climate scenario for RCP 8.5 by 2050.

Our research also reveals that tigers exhibit a preference for high‐altitude areas in response to these extreme events. Rising temperatures, altered precipitation patterns and the occurrence of floods and droughts are anticipated to disrupt the intricate food webs within the ecosystem. Consequently, these disruptions will have a negative impact on the herbivore prey, thereby affecting the tiger population across India, Bangladesh, Nepal, and Bhutan (Carter et al., 2013; Deb et al., 2019; Sanderson et al., 2010). Continued sightings of tigers at higher elevations in the eastern and western Himalayas further support the notion that tigers in the Indian subcontinent are highly adaptable, capable of thriving in diverse habitat types. These include saline mangrove forests in India (Roy et al., 2016), floodplains in Nepal (Thapa & Kelly, 2017) and even alpine forest habitats reaching elevations of up to 4400 metres in Bhutan (Tempa et al., 2019). Considering these findings, it becomes crucial to prioritize the assessment of climate changes, land use practices, and the expansion of tiger habitat in high‐altitude areas of the IEHR to safeguard the survival of this magnificent species.

Moreover, we found that broad‐leaved deciduous forests, grasslands, and forest cover or shrubland areas are the most suitable habitats for tigers and will host tiger habitat as indicated by Jhala et al. (2011). However, a previous study by Carter et al. (2013) argued that tigers are less likely to be found in areas with an abundance of riverine forests. Interestingly, our research contradicts this claim, as we have found that the river corridors and forests along the Brahmaputra River hold the greatest potential for tiger habitats (Borah et al., 2010), even under the most adverse climate scenarios. We have obtained significant records of tigers inhabiting the riverine islands, characterized by short and tall grasslands. Consequently, it is crucial to prioritize conservation efforts in these areas to safeguard the habitats of tigers.

The presence of rivers in the landscape plays a pivotal role in connecting existing habitats and signifies potential tiger habitats, as noted by Kywe (2012). The tributaries of the Brahmaputra River enable tiger movement across streams, facilitating activities such as hunting and promoting genetic diversity (Thatte et al., 2017). To ensure the protection of these vital habitats, it is imperative to establish conservation corridors along streams, as advocated by Stahl et al. (2020). In the floodplain areas of Assam, many protected areas (PAs) are interconnected by rivers, indicating a high likelihood of tiger occurrences on river islands that require protection from human activities, as highlighted by Borah et al. (2010). Therefore, conservation efforts in these regions hold significant importance for safeguarding the habitats of tigers.

Furthermore, we emphasize that to enhance our understanding of biodiversity conservation, particularly in relation to species' habitats, it is crucial to incorporate future land cover changes into climate impact assessments, as suggested by Ye et al. (2018). Relying solely on climatic variables for projecting future potential distributions could result in overprediction, making the integration of land cover changes essential for accurate assessments. In summary, these findings underscore the alarming impact of climate change and land cover change on tiger habitats, with changes in diet, loss of suitable habitat, and the combined threats of rising sea levels, extreme weather events, and human activities posing significant challenges to the survival of this iconic species. Urgent conservation measures and efforts to mitigate climate change are imperative to ensure the continued existence of tigers in these vulnerable ecosystems.

### 4.4 Conservation priority areas (CPs)

The conservation of tigers is a vital issue, and our study has revealed some crucial insights into their habitat in the IEHR. Our findings reveal that the CPs in this region are limited, comprising only 3.52% of the total land area. Interestingly, we have observed that tiger habitats are not solely restricted to PAs but are distributed across various forest patches. This aligns with earlier research indicating that a substantial proportion of tigers (35%) in India reside outside of PAs (Jhala et al., 2019). Our study further demonstrates that the potential habitat for tigers outside of PAs, encompassing high and highest priority areas, is more extensive compared to within PAs. Consequently, it is crucial to preserve tiger habitats beyond PAs to facilitate gene flow through migration corridors (Dutta et al., 2016; Natesh et al., 2017; Rautela et al., 2022; Thatte et al., 2017). However, our study also highlights the concerning fact that only 20.03% of the high‐priority areas for tiger conservation in the IEHR under protection, while a substantial 57.97% of the area outside PAs lacks wildlife protection (Wikramanayake et al., 2011). Despite this, we have observed that certain areas beyond the PA network, such as those inhabited by the indegenous Idu Mishmi community, exhibit a commitment to tiger conservation through their unique socio‐cultural values and taboos (Gopi et al., 2014). Hence, involving local communities in tiger conservation efforts can play a pivotal role in safeguarding the species and their habitats (Gubbi et al., 2008).

**FIGURE 10:**
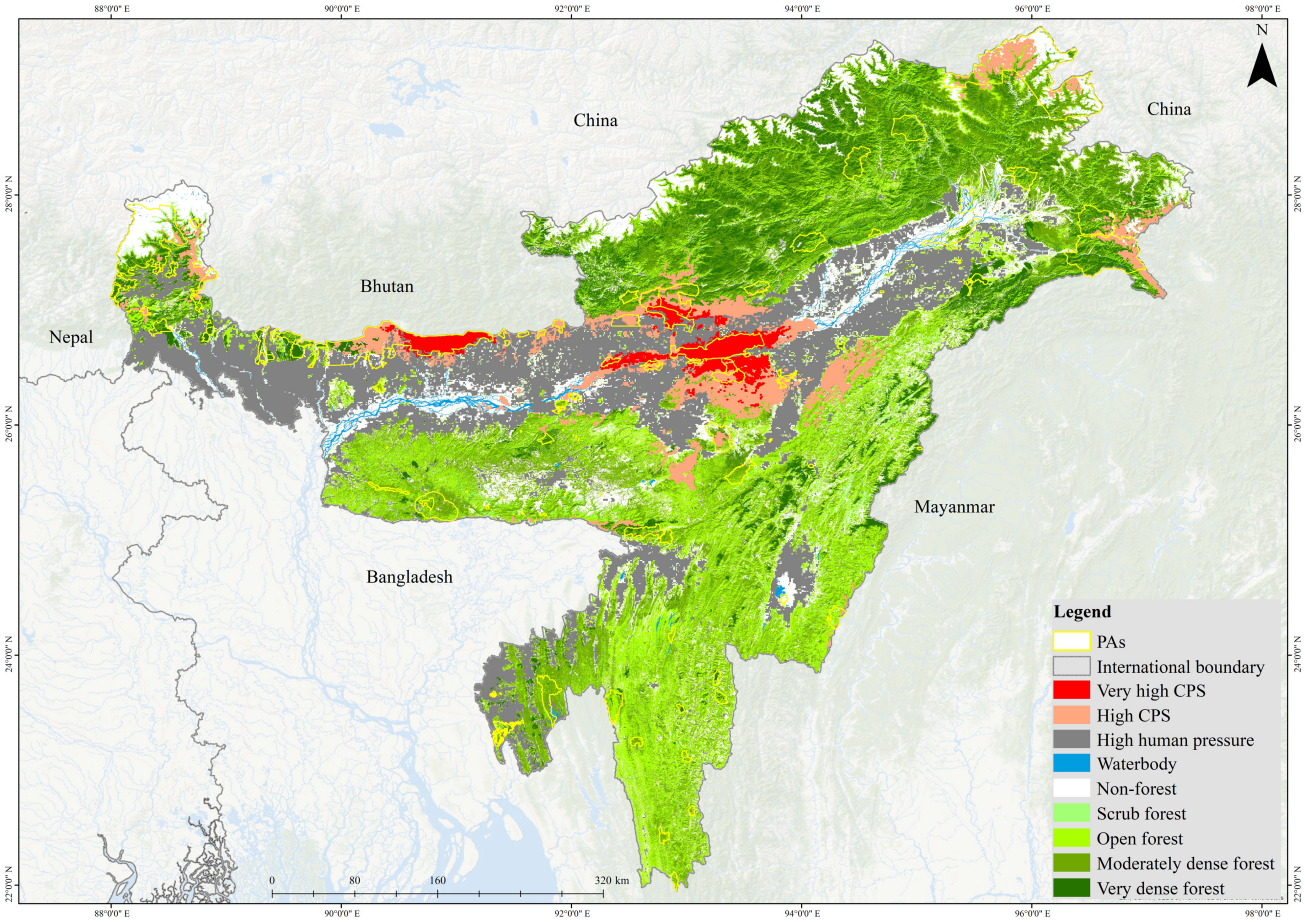
Recommended conservation priority sites for tiger in Indian East Himalayan Region. Protected areas (PAs) are demarcated with yellow boundary. Very high conservation priority areas (CPs) and high conservation priority areas (CPs) are need immediate attention for tiger conservation.

To ensure the preservation of genetic diversity, our study recommends the protection of highly prioritized areas, including community forests, the establishment of new protected areas or expansions of existing ones under the Wildlife Protection Act (Thatte et al., 2017). Key priority sites for tiger conservation in the IEHR encompass the KKAL, certain regions of Nagaland, the southern site of Yangoupokpi‐Lokchao Wildlife Sanctuary in Manipur, Namdapha Tiger Reserve and the eastern part of Pakke Tiger Reserve. These areas have relatively less human activity but are at risk of future deforestation and are currently not protected, except for PAs. Thus, efforts should be made to grant protected status to these vulnerable areas, as mentioned by Menon et al. (2001). However, it is concerning that the Manas‐Chirang landscape and the northern part of Orang National Park, which are also highly prioritized, face significant human disturbances. Overall, our study indicates that high‐potential areas for tiger habitat are characterized by low human pressure, proximity to riverine habitat, presence of bamboo forests, grasslands with high canopy cover, and mixed deciduous forests. Moreover, the presence of abundant prey in these areas is critical for their potential as tiger habitats. It is imperative to establish better connectivity for the free movement of wildlife in these regions (see Figure 10). Additionally, raising conservation awareness through programmes is essential to promote an understanding of the importance of tiger habitats in the context of climate change, particularly in the high‐altitude potential habitats of the IEHR (Mishra et al., 2006). Agroforestry practices can also contribute to the survival of tiger populations, especially in hilly areas, by protecting suitable habitats around PAs (Imron et al., 2011). Furthermore, conservation measures should extend beyond protected areas to encompass other potential habitats worthy of conservation efforts.

### 4.5 Threats to protected areas

The study reinforces that the areas of highest conservation priority for tigers in the IEHR are predominantly located within PAs. These PAs serve as healthy habitats, boasting forests and prey populations (Yumnam et al., 2014). Nevertheless, the study also sheds light on the challenges faced by these crucial habitats. Factors, such as forest fragmentation (Vergara et al., 2020), habitat degradation (Clark et al., 2013), natural resource exploitation (Lopoukhine et al., 2012), and high human pressure pose threats to these high‐priority habitats. Unfortunately, as many as 41 PAs are currently threatened from within, while 44 PAs are threatened from outside, making the matrix outside of PAs a critical area for conservation efforts (Stolton et al., 2015). It is, therefore, crucial to effectively manage habitats outside of PAs to meet global conservation goals and preserve biodiversity (Barnes et al., 2016). Additionally, habitat fragmentation (Dinerstein et al., 2007), poaching (Madhusudan & Karanth, 2002), and human–tiger conflict (Goodrich, 2010) pose significant threats to tiger populations, affecting their mating, food availability, and survival.

Tiger reserves and other protected areas play a crucial role in addressing threats to tigers and safeguarding potential habitats. However, our research demonstrates that Wildlife Sanctuaries in IEHR face greater threats compared to National Parks or Tiger reserves, primarily due to illegal entry and human settlements near these protected areas (Ogra, 2008). Agricultural activities have also emerged as a significant threat to mammal abundance in recent times, as evidenced in our study (de Lima et al., 2018).

Therefore, it is imperative to control human pressure resulting from unsustainable development and other activities in high‐potential areas. This is necessary to maintain fragmented landscapes and ensure the long‐term persistence of species through improved connectivity, facilitated by protected areas or forest corridors (Dutta et al., 2016; Thatte et al., 2017). With the increasing tiger population, the need for expanded habitat for their home range leads to fragmentation within protected areas, potentially triggering human–wildlife interactions as wildlife moves closer to human settlements (Majgaonkar et al., 2019). Hence, proactive management and planning for tiger habitats are essential to minimize the impacts of human activity and secure their long‐term survival.

Our study reveals that the assigned range map for tigers in the IEHR, as indicated by the IUCN Red List, is overestimated. This overestimation of range was also noted by Ramesh et al. (2017) for endemic birds in the Western Ghats. To obtain a more precise assessment of tiger status, it is crucial to update the range map using high‐resolution satellite data and SDM, as demonstrated in previous studies (Kaky & Gilbert, 2019; Syfert et al., 2014). We recommend that tiger reserves in the IEHR incorporate the current presence or range of tigers. Additionally, highly suitable areas that are currently categorized as extinct should be reconsidered and included in the IUCN extant category to enhance their global significance. Furthermore, further research is required to assess the status of uncertain areas, which may necessitate intensive sampling to determine the presence or absence of tigers. Accurately determining the distribution of tigers in the region allows for targeted conservation efforts in specific areas, improving their protection and management. Ultimately, this will contribute to the long‐term survival of tigers and the conservation of biodiversity in the IEH region.

## 5 CONCLUSIONS

The study confirms that potential tiger habitats outside of PAs are significant and need to be brought under the PAs network or as community reserves to ensure the representativeness and persistence of the ecosystem. Due to erratic temperatures and precipitation, potential tiger habitats are likely to shift to higher altitudes. However, their distribution is expected to decline under the worst climate and land use change scenarios. Therefore, restoring degraded forests in lower elevated areas is crucial. There is an urgent need for prevention environmentally unsustainable development projects that could have enormous impacts on tiger conservation in the IEHR. Conducting tiger and prey surveys, especially in the high‐potential habitats of PAs and outside of PAs, is also essential. We suggest IUCN tiger range needs to be evaluated using high‐resolution satellite data and SDM. Overall, this study provides baseline information that would help further research in implementing a connectivity study of the tiger population among the PAs in the Indo‐Burma and Eastern Himalayan biodiversity hotspots.

## AUTHOR CONTRIBUTIONS

**Jyotish Ranjan Deka:** Conceptualization (equal); data curation (equal); formal analysis (equal); methodology (equal); software (equal); validation (equal); writing – original draft (equal). **Sk. Zeeshan Ali:** Conceptualization (equal); data curation (equal); methodology (equal); validation (equal); visualization (equal); writing – original draft (equal). **Mujahid Ahmad:** Conceptualization (equal); data curation (equal); methodology (equal); validation (equal); visualization (equal); writing – original draft (equal). **Priyanka Borah:** Data curation (equal); writing – original draft (equal). **Govindan Veeraswami Gopi:** Writing – review and editing (equal). **Ruchi Badola:** Supervision (equal); writing – review and editing (equal). **Rabindra Sharma:** Writing – review and editing (equal). **Syed Ainul Hussain:** Conceptualization (equal); funding acquisition (equal); investigation (equal); supervision (equal); writing – review and editing (equal).

## CONFLICT OF INTEREST STATEMENT

The authors declare that they have no known competing financial interests or personal relationships that could have appeared to influence the work reported in this paper.

## Supporting information


Appendix S1.

## Data Availability

Data used in this article is available at (URL: https://doi.org/10.5061/dryad.8gtht76t8).
