# Supplementary material for: Can Bengal Tiger (Panthera tigris tigris) endure the future climate and land use change scenario in the East Himalayan Region? Perspective from a multiple model framework
Source: Ecol Evol. 2023 Aug 6;13(8):e10340. doi: 10.1002/ece3.10340 (PMC10404654; doi:10.1002/ece3.10340)
Supplement: Supplementary file 1 — Appendix S1. [file ECE3-13-e10340-s001.docx]

**Manuscript Number ECE-2023-03-00533.R1**

**Can Bengal Tiger (*Panthera tigris tigris*) endure the future climate and land use change scenario in the East Himalayan Region? Perspective from a multiple model framework**

**Appendix**

**S1; Variables for tiger in the Indian East Himalayan Region to evaluate the distribution model**

**Table S.1** List of Predicted variables with details.

| Variable | Environmental variable |
| --- | --- |
| BIO1 | Annual Mean Temperature |
| BIO2 | Mean Diurnal Range (Mean of monthly (max temp - min temp)) |
| BIO3 | Isothermality (BIO2/BIO7) (×100) |
| BIO4 | Temperature Seasonality (standard deviation ×100) |
| BIO5 | Max Temperature of Warmest Month |
| BIO6 | Min Temperature of Coldest Month |
| BIO7 | Temperature Annual Range (BIO5-BIO6) |
| BIO8 | Mean Temperature of Wettest Quarter |
| BIO9 | Mean Temperature of Driest Quarter |
| BIO10 | Mean Temperature of Warmest Quarter |
| BIO11 | Mean Temperature of Coldest Quarter |
| BIO12 | Annual Precipitation |
| BIO13 | Precipitation of Wettest Month |
| BIO14 | Precipitation of Driest Month |
| BIO15 | Precipitation Seasonality (Coefficient of Variation) |
| BIO16 | Precipitation of Wettest Quarter |
| BIO17 | Precipitation of Driest Quarter |
| BIO18 | Precipitation of Warmest Quarter |
| BIO19 | Precipitation of Coldest Quarter |
| Forest type | Forest cover |
| NDVI | Normalized Difference Vegetation Index |
| Tree canopy | Tree canopy |
| LULC | Land cover |
| Ele | Elevation |
| Slope | Slope |
| Ecu roads | Euclidian distance to roads |
| Ecu rivers | Euclidian distance to river |
| Human modification | Human modification |
| Human footprint | Human footprint |


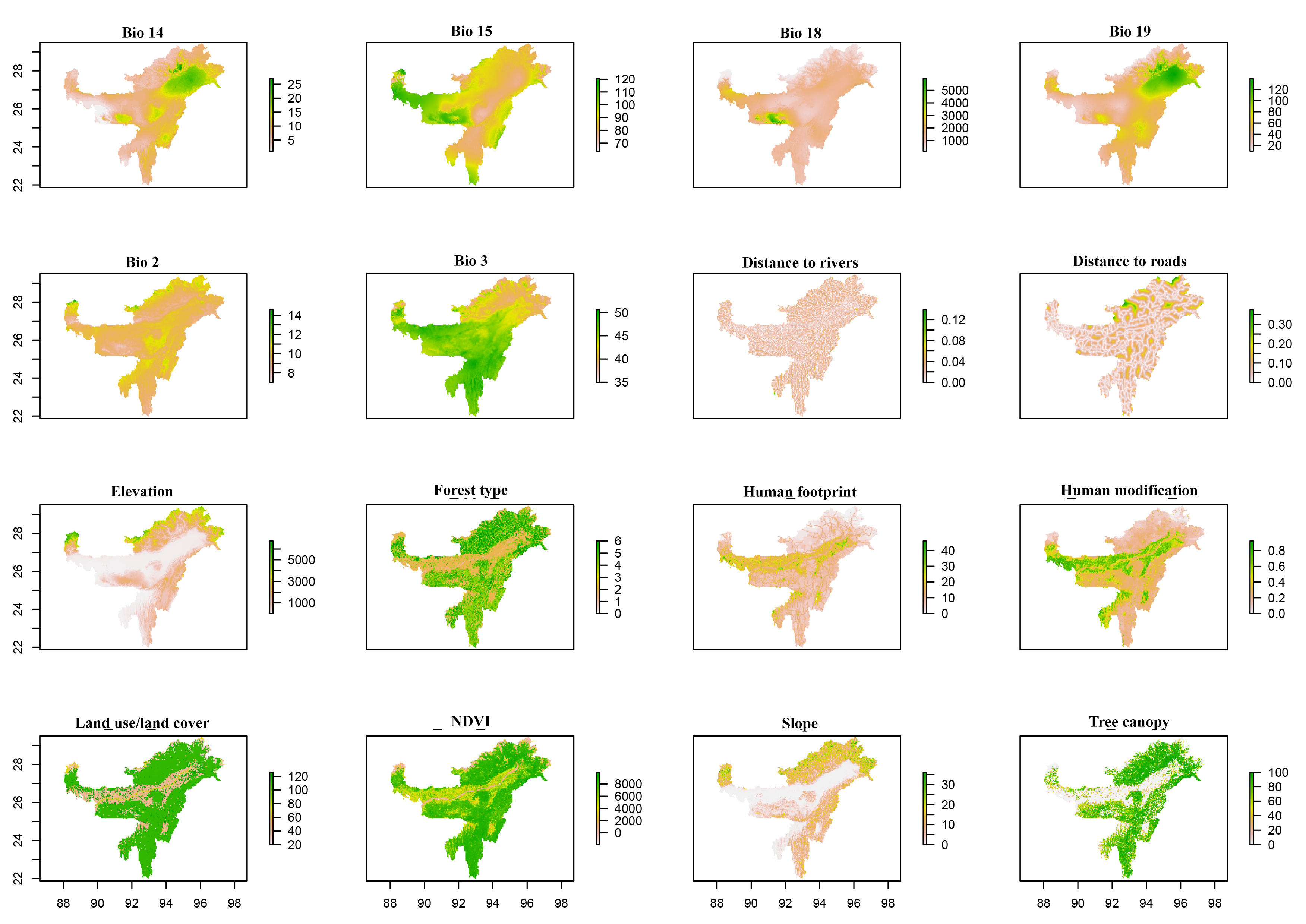


**Fig. S1:** Predicted variables for tiger in the Indian East Himalayan Region to evaluate the distribution model.

**S2; Relative variable importance for each independent SDM model and response curve for current baseline model**


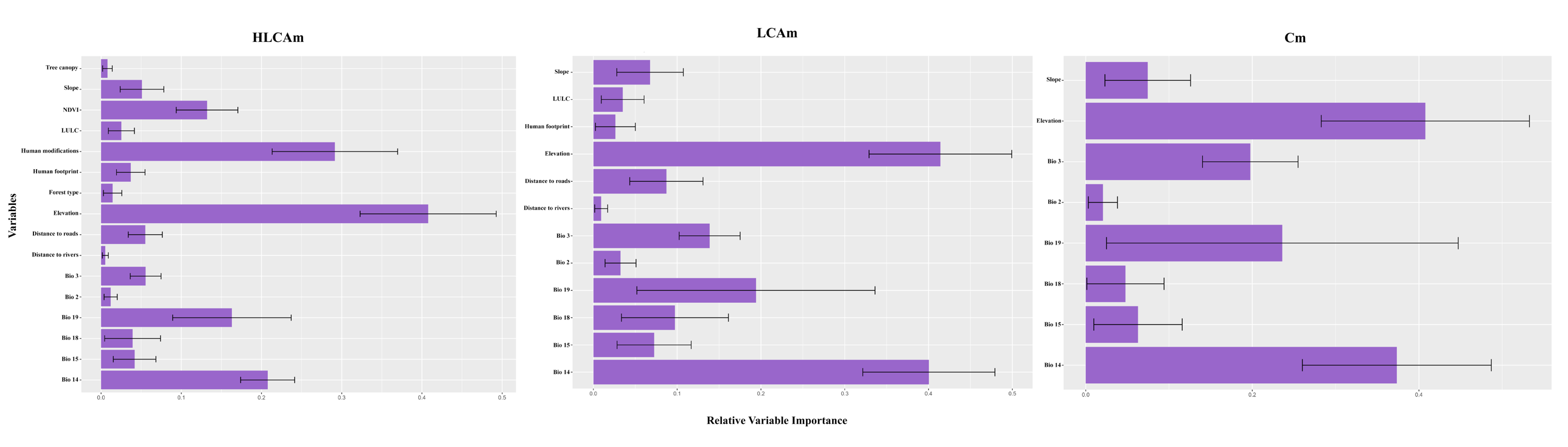


**Fig. S2.1:** Relative variable importance for each independent SDM model





**Fig. S2.2:** The marginal response curve for all the variables used for the model prediction, **a.** Bio2, **b.** Bio3, **c.** Bio14, **d.** Bio15, **e.** Bio18, **f.** Bio19, **g.** Distance to roads, **h.** Distance to rivers**, i.** Elevation, **j.** Forest type, **k.** Human footprint, **l.** Human modification, **m.** LULC, **n.** NDVI, **o.** Slope, **p.** Tree canopy.

**S.3: ODMAP protocol**

Can Bengal Tiger (*Panthera tigris tigris*) endure the future climate and land use change scenario in the East Himalayan Region? Perspective from a multiple model framework

2023-03-07

## Overview

#### Authorship

#### Model objective

Model objective: Mapping and interpolation

Target output: Continuous habitat suitability index and conservation priority areas

#### Focal Taxon

Focal Taxon: *Panthera tigris tigris*

#### Location

Location: Indian East Himalaya Region

#### Scale of Analysis

Spatial extent: 87.98722889, 97.41204028, 21.94063778, 29.46155278 (xmin, xmax, ymin, ymax)

Spatial resolution: 1km

Temporal extent: Recent (2000-2022)

Boundary: natural, political

#### Biodiversity data

Observation type: field survey, standardised monitoring data, range map, literature review

Response data type: presence-only

#### Predictors

Predictor types: climatic, habitat, topographic, Land use

#### Hypotheses

Hypotheses: Land cover, climate, habitat type, and terrain are correlated with tiger distribution in the Indian East Himalayan Region.

#### Assumptions

Model assumptions:

1. The only climate variables SDM overpredicted the species range under future climate regimes.

2. Species–environment equilibrium, Availability of all important predictors.

3. Relevant ecological drivers (or proxies) of species distributions are included.

#### Algorithms

Modelling techniques: maxent, glm, brt, randomForest, mars

Model complexity: In order to model the fundamental niche of the species, we use an ensemble of statistical and machine learning algorithms that fit smooth and/or complex response surfaces.

Model averaging: Weighted mean ensemble of AUC

#### Workflow

Model workflow: “see Workflow diagram”

#### Software

Software: R (4.1.3 version), ArcGIS ver. 10.8.2, QGIS ver. 3.22.3, MS excel 2019

## Data

#### Biodiversity data

Taxon names: *Panthera tigris tigris*

Taxonomic reference system: https://www.itis.gov/servlet/SingleRpt/SingleRpt?search_topic=TSN&search_value=183805#null

Ecological level: species

Data sources:

Sampling design: Random

Sample size: *Panthera tigris tigris* (1471)

Spatial thinning: Using the spThin-package in R and a distance between pixels of 5km to account for auto-correlation.

Absence data: NA

Background data: Generation of pseudo-absences by sampling points outside a 5 km buffer from the presence records.

#### Data partitioning

Training data: 70% training data and 30% testing data; Absences were randomly subsampled to match presence counts (BRT, with 10 runs)

Validation data: Model fitted using 5-fold cross validation

#### Predictor variables

Predictor variables: CHELSA bioclimatic variables; 19 bioclimatic variable, Land cover; Grassland, woodland, urban, waterbody, and shrubs, Anthropogenic activities; distance from roads, human footprint, and human modification; Habitat; tree cover, forest type, NDVI, and distance from waterbody, Topography; elevation.

Data sources:

Climate: CHELSA bioclimatic variables ( <https://chelsa-climate.org/>)

Land cover: Global land-use and land cover change (LUCC) simulation product at a 1-km resolution using GeoSOS-FLUS ( <http://geosimulation.cn/GlobalLUCCProduct.html>).

Anthropogenic activities:

distance from roads; Distance from road was created from the current road network.

human footprint; (<https://sedac.ciesin.columbia.edu/data/set/wildareas-v3-2009-human-footprint>)

human modification; (<https://sedac.ciesin.columbia.edu/data/set/lulc-human-modification-terrestrial-systems>).

Habitats:

Tree cover: (<http://apps.worldagroforestry.org/global-tree-cover/data-download.html>)

Forest type: Forest Survey of India, 2019 (<https://fsi.nic.in/>)

NDVI: Normalized Difference Vegetation Index (NDVI). (<https://www.ncei.noaa.gov/products/climate-data-records/normalized-difference-vegetation-index>).

distance from waterbody: Distance from waterbody was created from the river network.

Topography: Elevation (<http://www.worldclim.org/>).

Spatial extent: 87.98722889, 97.41204028, 21.94063778, 29.46155278 (xmin, xmax, ymin, ymax)

Spatial resolution: 1km

Coordinate reference system: WGS 84 /UTM zone 46N (EPSG:32646)

Temporal extent: 1990-2019

#### Transfer data

Data sources:

CHELSA bioclimatic variables (19 predictors): CHELSA bioclimatic variables of Community Climate System Model (CCSM5). ( <https://chelsa-climate.org/>)

Land use scenario: Global land use and land cover change simulations for the years 2050 from the GeoSOS global database to project future scenarios for human land use changes (Liu et al. 2017).

Spatial extent: 87.98722889, 97.41204028, 21.94063778, 29.46155278 (xmin, xmax, ymin, ymax)

Spatial resolution: 1km

Temporal extent: (2041-2060)

Models and scenarios: Community Climate System Model, RCP 4.5 and RCP 8.5; We included land use change scenarios of A1B (moderate increase in land use across all resources).

Quantification of Novelty: NA

## Model

#### Multicollinearity

Multicollinearity: We performed by variance inflation factor (or VIF) in R (Graham 2003; Naimi and Araújo, 2016).

#### Model settings

Maxent: We enabled the use of all six feature classes (linear, product, quadratic, hinge, threshold and categorical) for modelling species responses to environmental variables. The default value of 1.0 was used as the regularization parameter, which affects how closely the training data is fitted.

Glm: GLMs were fitted with linear and quadratic terms with equal weighting

Brt: BRTs were estimated with a tree complexity of 2, a bag fraction of 0.75 and a variable learning rate such that 1000- 5000 trees were fitted (Elith et al., 2008).

randomForest: Random forests were fitted with 1000 trees, and a minimum nodesize of 20.

Mars: MARS were fitted with two level interactions between predictors.

Model settings (extrapolation): Model settings were chosen to map the potential distribution within the study area. We do not attempt to extrapolate to other areas.

#### Model estimates

#### We analysed model coefficients by comparing multiple SDM models

#### Analysis and Correction of non-independence

Spatial autocorrelation: NA

#### Threshold selection

Threshold selection: Minimum threshold presence was used for creating a binary map

#### Performance statistics

Performance on training data: The averaged AUC and TSS scores were used as model predictive performance measures, following a 5-fold cross-validation procedure with ten replicates.

Performance on validation data: The averaged AUC and TSS scores were used as model predictive performance measures, following a 5-fold cross-validation procedure with

Performance on test data: Boyce Index

#### Plausibility check

Response shapes: Partial dependence plots

Expert judgement: Inspecting projected distribution

## Prediction

#### Prediction output

Prediction unit: Continuous occurrence probability and potential presence.

#### Uncertainty quantification

Novel environments: Multiple SDM will help to choose best projected model to use for conservation planning.
